# Polygenic liabilities and treatment trajectories in early-onset depression: a Danish register-based study

**DOI:** 10.1017/S0033291724002186

**Published:** 2024-10

**Authors:** Jessica Mundy, Alisha S. M. Hall, Jette Steinbach, Clara Albinaña, Esben Agerbo, Thomas D. Als, Anita Thapar, John J. McGrath, Bjarni J. Vilhjálmsson, Merete Nordentoft, Thomas Werge, Anders Børglum, Preben B. Mortensen, Katherine L. Musliner

**Affiliations:** 1 Department for Clinical Medicine, Aarhus University, Aarhus, Denmark; 2 National Centre for Register-based Research, Aarhus University, Aarhus, Denmark; 3 The Lundbeck Foundation Initiative for Integrative Psychiatric Research (iPSYCH), Denmark; 4 Department of Biomedicine, Aarhus University, Aarhus Denmark; 5 Center for Genomics and Personalized Medicine (CGPM), Aarhus, Denmark; 6 Wolfson Centre for Young People's Mental Health, Centre for Neuropsychiatric Genetics and Genomics, Division of Psychological Medicine and Clinical Neuroscience, Cardiff University, UK; 7 Queensland Brain Institute, University of Queensland, Brisbane, QLD, Australia; 8 Queensland Centre for Mental Health Research, The Park Centre for Mental Health, Wacol, 4076, Australia; 9 Bioinformatics Research Centre (BIRC), Aarhus University, Aarhus Denmark; 10 Novo Nordisk Foundation Center for Genomic Mechanisms of Disease, The Broad Institute of MIT and Harvard, MA, USA; 11 Copenhagen Research Center for Mental Health (CORE), Mental Health Center Copenhagen, Mental Health services in the Capital Region of Denmark; 12 Department of Clinical Medicine, Faculty of Health Sciences, University of Copenhagen, Denmark; 13 Institute of Biological Psychiatry, Copenhagen Mental Health Services, Copenhagen, Denmark; 14 Department for Affective Disorders, Aarhus University Hospital-Psychiatry, Aarhus, Denmark

**Keywords:** depression, major depressive disorder, genetics, polygenic score, treatment, trajectory, secondary care, antidepressants

## Abstract

**Background:**

The clinical course of major depressive disorder (MDD) is heterogeneous, and early-onset MDD often has a more severe and complex clinical course. Our goal was to determine whether polygenic scores (PGSs) for psychiatric disorders are associated with treatment trajectories in early-onset MDD treated in secondary care.

**Methods:**

Data were drawn from the iPSYCH2015 sample, which includes all individuals born in Denmark between 1981 and 2008 who were treated in secondary care for depression between 1995 and 2015. We selected unrelated individuals of European ancestry with an MDD diagnosis between ages 10–25 (*N* = 10577). Seven-year trajectories of hospital contacts for depression were modeled using Latent Class Growth Analysis. Associations between PGS for MDD, bipolar disorder, schizophrenia, ADHD, and anorexia and trajectories of MDD contacts were modeled using multinomial logistic regressions.

**Results:**

We identified four trajectory patterns: *brief contact* (65%), *prolonged initial contact* (20%), *later re-entry* (8%), and *persistent contact* (7%). Relative to the *brief contact* trajectory, higher PGS for ADHD was associated with a decreased odds of membership in the *prolonged initial contact* (odds ratio = 1.06, 95% confidence interval = 1.01–1.11) and *persistent contact* (1.12, 1.03–1.21) trajectories, while PGS-AN was associated with increased odds of membership in the *persistent contact* trajectory (1.12, 1.03–1.21).

**Conclusions:**

We found significant associations between polygenic liabilities for psychiatric disorders and treatment trajectories in patients with secondary-treated early-onset MDD. These findings help elucidate the relationship between a patient's genetics and their clinical course; however, the effect sizes are small and therefore unlikely to have predictive value in clinical settings.

## Introduction

Major depressive disorder (MDD) is a common, burdensome psychiatric disorder with a lifetime prevalence of up to 20% (Alonso et al., 2004; Andrade et al., 2003; Hasin et al., 2018; Kessler & Bromet, 2013). In Denmark, the most severe, complex cases of MDD are treated by psychiatrists in hospital-based settings (i.e. secondary care), which includes inpatient, outpatient, and visits to emergency departments. Lifetime risks for diagnosis of MDD in this setting are 16% for women and 9% for men (Pedersen et al., 2014). The clinical course of MDD is highly variable: some individuals experience a single episode before recovering fully, others experience recurrent episodes punctuated by periods of recovery, and still others experience chronic symptoms (Eaton et al., 2008). In a previous study, Musliner et al. (2016) found that most MDD patients in Danish psychiatric hospitals (77%) exited secondary treatment within 2 years. What happened to these patients – whether they continued MDD treatment in primary care, transitioned to a different psychiatric diagnosis, or fully recovered – is unknown. Of the remaining patients, 13% exhibited prolonged initial contact, 7% quickly exited secondary care but re-entered later, and a small minority (3%) had persistent contact for at least 10 years.

Compared to individuals diagnosed later in life, those with early-onset MDD are more likely to experience greater illness severity, decreased social and occupational function, medical and psychiatric comorbidity, suicide attempts, and lower quality of life (Hakulinen, Musliner, & Agerbo, 2019; McGrath et al., 2020; Zisook et al., 2007). These patients therefore represent a particularly vulnerable group for whom early prediction of prognostic course would be useful. If identified early, these patients could potentially be given more intensive treatment or followed more closely. Additionally, early identification could help target individuals most in need of social and occupational support. Targeted interventions could potentially improve education and employment prospects for vulnerable individuals (López-López et al., 2019; Plana-Ripoll et al., 2022).

There is growing recognition that the clinical course of MDD is partly influenced by genetics. Family studies show that the heritability is higher among probands with recurrent depression (Bland, Newman, & Orn, 1986; McGuffin, Katz, Watkins, & Rutherford, 1996; Sullivan, Neale, & Kendler, 2000), and that having a parent with MDD increases risk for more severe and recurrent forms of the disorder (Lieb, Isensee, Höfler, Pfister, & Wittchen, 2002). Recently, two genome-wide association studies (GWASs) using UK Biobank data showed that MDD's common variant (i.e. SNP-based) heritability is slightly larger among individuals with recurrent episodes compared to those with single-episode MDD, although the genetic overlap between the two is high (*r*_g_ = 0.85–0.94) (Coleman, Gaspar, Bryois, & Breen, 2020; Nguyen et al., 2022). More convincing evidence of genetic heterogeneity comes from comparisons of mild vs severe forms of MDD, where the genetic overlap is around 0.65 (Nguyen et al., 2022).

The output of a GWAS can be used to calculate a composite measure of an individual's genetic liability, known as a polygenic score (PGS) (Choi, Mak, & O'Reilly, 2020). Wray et al. (2018) showed that PGS-MDD was, on average, higher among individuals with recurrent compared to single-episode MDD (Wray et al., 2018). Studies have also found associations between higher PGS-MDD and number of depressive episodes (Mitchell et al., 2022) and risk for recurrence (Als et al., 2023; Musliner et al., 2021). These findings suggest that PGSs could become useful for predicting a patient's clinical trajectory at their first episode.

Our main goal was to examine the associations between PGSs for six major psychiatric disorders (MDD, bipolar disorder [BD], schizophrenia [SCZ], attention-deficit-hyperactivity disorder [ADHD], autism spectrum disorder [ASD], and anorexia nervosa [AN]) and long-term treatment trajectories in patients with early-onset MDD in secondary care. We focused on early-onset MDD (defined as an MDD diagnosis by age 25) because members of this group are at heightened risk of poor course and outcomes and therefore deserve special attention. As past research has shown that the majority of patients exit secondary care within 1–2 years (Musliner et al., 2016) we examined other indicators of 7-year treatment trajectories in this group, including continued medication treatment in primary care and secondary treatment for other psychiatric disorders. As secondary aims, we compared the effects of PGS to parental history, another indicator of genetic liability, and examined whether the PGS associations differed based on age at first MDD diagnosis.

## Methods

### Data source

Data were obtained from the Lundbeck Foundation Initiative for Integrative Psychiatric Research 2015 case-cohort sample (iPSYCH2015) (Bybjerg-Grauholm et al., 2020), which includes all individuals born between 1981 and 2008 who were diagnosed with six major psychiatric disorders (MDD, BD, SCZ, ADHD, ASD, and AN), in Danish psychiatric hospitals between 1995 and 2015 (Bybjerg-Grauholm et al., 2020; Pedersen et al., 2018). Psychiatric patients were identified from The Danish Psychiatric Central Research Register (DPCRR) which contains information on admission and discharge dates as well as diagnoses for all inpatient contacts at psychiatric hospitals in Denmark since 1969 and all inpatient, outpatient, and emergency contacts since 1995 (Mors, Perto, & Mortensen, 2011). Diagnoses are assigned at discharge by the treating psychiatrist based on the ICD-8 from 1969 to 1994 and the ICD-10 from 1995 onwards. Information on antidepressant prescriptions was obtained from The Danish National Prescription Register, which contains information on all prescriptions redeemed at Danish community pharmacies from 1995 to 2016 (Kildemoes, Sørensen, & Hallas, 2011). Demographic information was obtained from the Danish Civil Registration System (CPR) (Pedersen, 2011). Parental history of MDD, BD and SCZ spectrum disorders was extracted by linking information on parents from the CPR to information on psychiatric admissions in the DPCRR. Data linkage between registers was facilitated by the CPR number, a unique identification number assigned to all individuals living in Denmark.

The iPSYCH study was approved by the Scientific Ethics Committee in the Central Denmark Region and the Danish Data Protection Agency. In accordance with Danish legislation, the Danish Scientific Ethics Committee has, for this study, waived the need for specific informed consent in biomedical research based on existing biobanks.

### Genotyping

DNA was obtained from blood samples collected at birth as part of routine testing for congenital disorders and stored as dried blood spots in the Danish Newborn Screening Biobank. The first wave of iPSYCH (iPSYCH2012) was genotyped using the Illumina Infinium Psych chip v1.0 array (Pedersen et al., 2018) and the second wave (iPSYCH2015i) was genotyped with the Illumina Global Screening array (Bybjerg-Grauholm et al., 2020). In both samples, genotyping complied with the manufacturer's guidance.

### Sample selection

Online Supplemental Figure 1 contains a flowchart detailing the sample selection process. We selected all individuals from the iPSYCH2015 sample who received their first diagnosis of MDD (ICD-10 codes F32–F33) in secondary care (inpatient, outpatient, or emergency) between ages 10–25 years. We removed individuals who died or emigrated within 7 years of their initial diagnosis, as well as individuals diagnosed less than 7 years before 31st December 2016. We chose this as the administrative censoring date because it was the last date for which complete information on antidepressant prescriptions was available. We also removed individuals with a prior BD (ICD-10 codes F30–F31) or SCZ spectrum disorder (ICD-10 codes F2) diagnosis, or without available genetic data. We selected a homogenous ancestry group by first generating principal components (PCs) using the autoSVD algorithm from the R package bigsnpr (Privé, Luu, Blum, McGrath, & Vilhjálmsson, 2020) and then using the function dist_ogk from the bigutilsr R package (Privé et al., 2020) to retain individuals <4.8log(dist) units from the centre of the 20 PCs. Finally, we computed KING-relatedness robust coefficients and excluded at random one individual from each pair with >3rd degree relatedness. The final study sample included 10 577 individuals.

### Measures

We defined an MDD episode as any inpatient, outpatient, or emergency contact with an ICD-10 code of F32 or F33. Contacts occurring within 8 weeks of each other were collapsed into a single episode. The severity of the index episode was defined based on the diagnostic code (i.e. F3*.0 = mild, F3*.1 = moderate, F3*.2 = severe, F3*.3 = severe with psychotic symptoms). For index episodes that included multiple hospital contacts, individuals were categorized based on the contact with the highest severity, with ‘mild’ considered the least severe and ‘severe with psychotic symptoms’ considered the most severe. Similarly, the treatment setting of index episodes that included multiple contacts were categorized based on the most ‘severe’ setting out of all the contacts, with inpatient considered the most severe and outpatient the least severe. The length of the index episode was defined as the number of days between the start date of the first hospital contact and the discharge date of the last hospital contact within that episode.

PGSs for the 6 primary psychiatric outcomes studied in iPSYCH were calculated using a MetaPRS method (Albiñana et al., 2021) which uses both individual-level genetic data and summary statistics from external GWAS to increase the training effective sample size. This method has been shown to improve prediction for psychiatric outcomes particularly when the dataset is large (Albiñana et al., 2021). In this approach, an externally trained PGS calculated using the LDpred2 software (Vilhjalmsson et al., 2015) is combined with an internally trained PGS calculated using the BOLT-LMM software (Loh et al., 2015). These component scores are then combined into a metaPRS using a weighted linear regression:
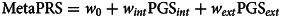


To train the external component, we used GWAS summary statistics from the Psychiatric Genomics Consortium's meta-analyses (Demontis et al., 2019; Grove et al., 2019; Mullins et al., 2021; Schizophrenia Working Group of the Psychiatric Genomics Consortium, 2014; Watson et al., 2019; Wray et al., 2018) excluding the iPSYCH samples. To train the internal component, we selected SNPs from the iPSYCH genotyped set where minor allele frequency >1% and missing values <10%. The training model was based on a sample of unrelated iPSYCH participants of European ancestry, from which was estimated the per-SNP prediction *β*s (BLUP). Additional covariates in the model included genotype wave, sex, age, and the first two principal components. Ten-fold cross-validation was used to avoid overfitting. The final combined metaPGSs were standardized within the study sample such that mean = 0 and standard deviation (s.d.) = 1.

### Statistical analysis

Trajectory patterns of hospital contacts were modeled using Latent Class Growth Analysis (LCGA) (Nagin, 2014; Nagin & Odgers, 2010) in SAS PROC TRAJ (Jones & Nagin, 2007). For each patient, we created a series of dummy variables indicating whether that patient was in contact with the secondary psychiatric system (yes/no) for MDD during successive 6-month time windows spanning the 7-year period following the initial MDD episode. We then fit a series of LCGA logit models with 1–6 classes in which the outcome of interest was the log odds of having a hospital contact for MDD during each 6-month interval. We included up to cubic polynomial terms for trajectory slopes.

For each PGS and family history variable, we calculated the odds ratio (OR), 95% confidence intervals (CIs), and *p*-value for trajectory class membership relative to the *brief contact* class using multinomial regression models adjusted for sex, age-at-onset, and calendar year-at-diagnosis in three-year bands. For the models with the PGSs as explanatory variables, we included the first five PCs and iPSYCH original cohort (iPSYCH2012 vs iPSYCH2015i) as additional covariates. Multinomial regressions were calculated in R version 4.1.1 using the multinom function from the nnet package. In addition, we fit multivariable regression with all 6 PGS variables in the same model to estimate the independent associations between each PGS and trajectory class membership.

For individuals in the trajectory class with early exit from the secondary treatment system, we examined the following outcomes during the remaining follow-up period: (1) subsequent medical treatment in primary care, defined as redeeming an antidepressant prescription (ATC codes N06A excluding N06AX12, which is only used for smoking cessation in Denmark (Skovlund, Mørch, Kessing, & Lidegaard, 2016)) and (2) a hospital-based diagnosis of one of the following: mental and behavioral disorder due to psychoactive substance use (i.e. substance abuse; ICD-10 codes in the F1 category), SCZ, schizotypal and delusional disorder (i.e. SCZ spectrum disorders; F2), neurotic, stress-related and somatoform disorders (i.e. anxiety disorders; F4) personality disorders (F6), and behavioral and emotional disorders with onset in childhood and adolescence (i.e. childhood/adolescent-onset disorders; F90–F98). For each PGS, we performed logistic regressions to calculate the OR, 95% CIs, and *p*-value for each outcome. The same covariates were included as per the multinomial regressions. Logistic regressions were calculated using the glm function in R.

Clinical guidelines in Denmark recommend that antidepressant treatment be extended for 6–12 months after an MDD episode to reduce risk of relapse (Sundhedsstyrelsen, 2007). Therefore, we also examined associations between each PGS – both separately and in a mutually adjusted multivariable model – and parental history variable and antidepressant prescriptions redeemed at least 6 months and 1 year after final discharge. In doing this, we hoped to capture outcomes that did not simply reflect features of the last MDD episode that brought them into secondary care.

In addition to modeling PGSs as continuous variables, we also fit models comparing the top 10% of each PGS distribution to the bottom 10%, 50%, and 90% (results from additional models comparing the top 5% of the distribution to the bottom 5%, 50%, and 95% are presented in the supplement). Finally, we examined the impact of age-at-onset by running separate models for individuals first diagnosed at 10–15, 16–20, and 21–25 years. Due to reduced sample size, calendar year at index episode was included as a binary variable (1995–2002, 2003–2009) in the age-stratified models.

Statistical significance was assessed at the Bonferroni-adjusted alpha of 0.0083 for the PGS (0.05/6 PGSs), and 0.017 for parental history (0.05/3 parental history variables).

## Results

### Sample characteristics

Sample characteristics for the full sample and by trajectory class are shown in Table 1. Seventy-one per cent of the sample was female. The median age at first MDD contact was 19 years (IQR = 5). Most of the sample received outpatient treatment at the index MDD episode (60%).

**Table 1.** Sample characteristics

	Overall sample	Trajectory class			
Characteristic	*N* = 10577^a^	Brief contact, N = 6876^a^	Prolonged initial contact, *N* = 2146^a^	Later re-entry, *N* = 845^a^	Persistent contact, *N* = 710^a^
Gender					
Female	7453 (70%)	4681 (68%)	1565 (73%)	668 (79%)	539 (76%)
Male	3124 (30%)	2195 (32%)	581 (27%)	177 (21%)	171 (24%)
Age at index episode (years)	19.0 (5.0)	19.0 (5.0)	18.0 (5.0)	19.0 (5.0)	17.0 (6.0)
Age at index episode (years) categorized					
10–15 years	2170 (21%)	1240 (18%)	518 (24%)	169 (20%)	243 (34%)
16–20 years	5165 (49%)	3420 (50%)	1052 (49%)	418 (49%)	275 (39%)
21–25 years	3242 (31%)	2216 (32%)	576 (27%)	258 (31%)	192 (27%)
Total no. episodes					
1	8083 (76%)	6124 (89%)	1430 (67%)	159 (19%)	370 (52%)
2	1887 (18%)	703 (10%)	550 (26%)	435 (51%)	199 (28%)
3+	607 (5.7%)	49 (0.7%)	166 (7.7%)	251 (30%)	141 (20%)
Length of index treatment period (days)					
Less than 1 day	2274 (21%)	1971 (29%)	100 (4.7%)	173 (20%)	30 (4.2%)
1 day	397 (3.8%)	334 (4.9%)	23 (1.1%)	31 (3.7%)	9 (1.3%)
2–30 days	1125 (11%)	928 (13%)	68 (3.2%)	101 (12%)	28 (3.9%)
31–60 days	674 (6.4%)	551 (8.0%)	49 (2.3%)	58 (6.9%)	16 (2.3%)
61–90 days	616 (5.8%)	527 (7.7%)	46 (2.1%)	35 (4.1%)	8 (1.1%)
91–120 days	595 (5.6%)	482 (7.0%)	34 (1.6%)	64 (7.6%)	15 (2.1%)
120 days+	4896 (46%)	2083 (30%)	1826 (85%)	383 (45%)	604 (85%)
Treatment setting at index episode					
Inpatient	2287 (22%)	1302 (19%)	548 (26%)	204 (24%)	233 (33%)
Outpatient	6349 (60%)	3890 (57%)	1508 (70%)	502 (59%)	449 (63%)
Emergency	1941 (18%)	1684 (24%)	90 (4.2%)	139 (16%)	28 (3.9%)
Severity at index episode					
Mild	2041 (19%)	1427 (21%)	352 (16%)	177 (21%)	85 (12%)
Moderate	4668 (44%)	2968 (43%)	1018 (47%)	363 (43%)	319 (45%)
Severe	1158 (11%)	609 (8.9%)	311 (14%)	116 (14%)	122 (17%)
Severe with psychotic symptoms	447 (4.2%)	245 (3.6%)	110 (5.1%)	35 (4.1%)	57 (8.0%)
Severity not specified	2263 (21%)	1627 (24%)	355 (17%)	154 (18%)	127 (18%)
Parental history of MDD	1723 (16%)	1106 (16%)	330 (15%)	169 (20%)	118 (17%)
Parental history of mania/BD	307 (2.9%)	210 (3.1%)	46 (2.2%)	31 (3.7%)	20 (2.8%)
Parental history of SCZ spectrum	387 (3.7%)	247 (3.6%)	71 (3.3%)	33 (3.9%)	36 (5.1%)

Sample included individuals who had been treated for major depressive disorder (MDD) in a Danish psychiatric hospital between ages of 10 and 25. Trajectory classes were identified using Latent Class Growth Analysis and were based on hospital contacts for MDD over a 7-year period

a n (%); Median (IQR)MDD, major depressive disorder; BD, bipolar disorder; SCZ, schizophrenia.

### PGS and trajectory patterns in secondary care

**Figure 1.**
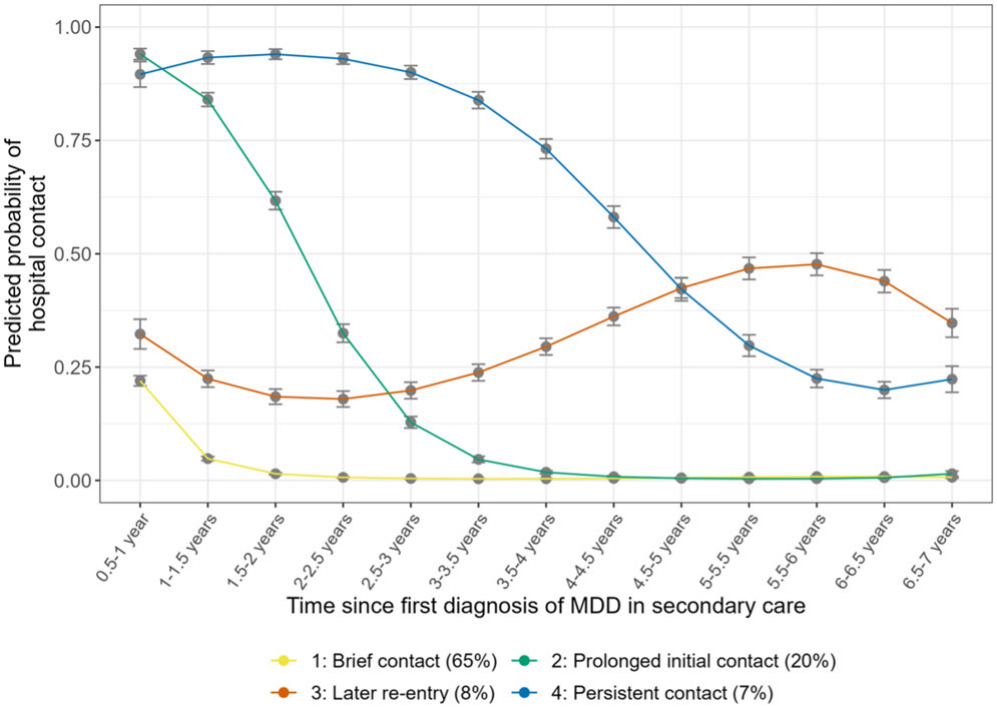
Course trajectory patterns in the 4-class model. Trajectory classes were identified by Latent Class Growth Analysis and were based on hospital contacts for major depressive disorder (MDD) over a 7-year period.

Trajectory patterns for models with 1–6 classes are shown in online Supplemental Figure 2**.** We selected the 4-class model (Fig. 1) as the final model because it represented the best balance of statistical fit, parsimony, and clinical utility (online Supplemental Table 1). The largest class containing 65% of the sample had a trajectory characterized by *brief contact* (class 1). These individuals exited secondary treatment within 2 years after initial hospital contact, most within 6 months. The next largest class (20%) included those who showed *prolonged initial contact* (class 2). These individuals had a high, decreasing probability of contact for 4 years after their initial contact, and then did not return during the remaining follow-up. Around 8% of the sample exited early but then had *later re-entry* into secondary care (class 3). The smallest group (7%) had *persistent contact*, with 75–90% probability of contact in years 1–4 decreasing to ~25% in years 5–7.

**Figure 2.**
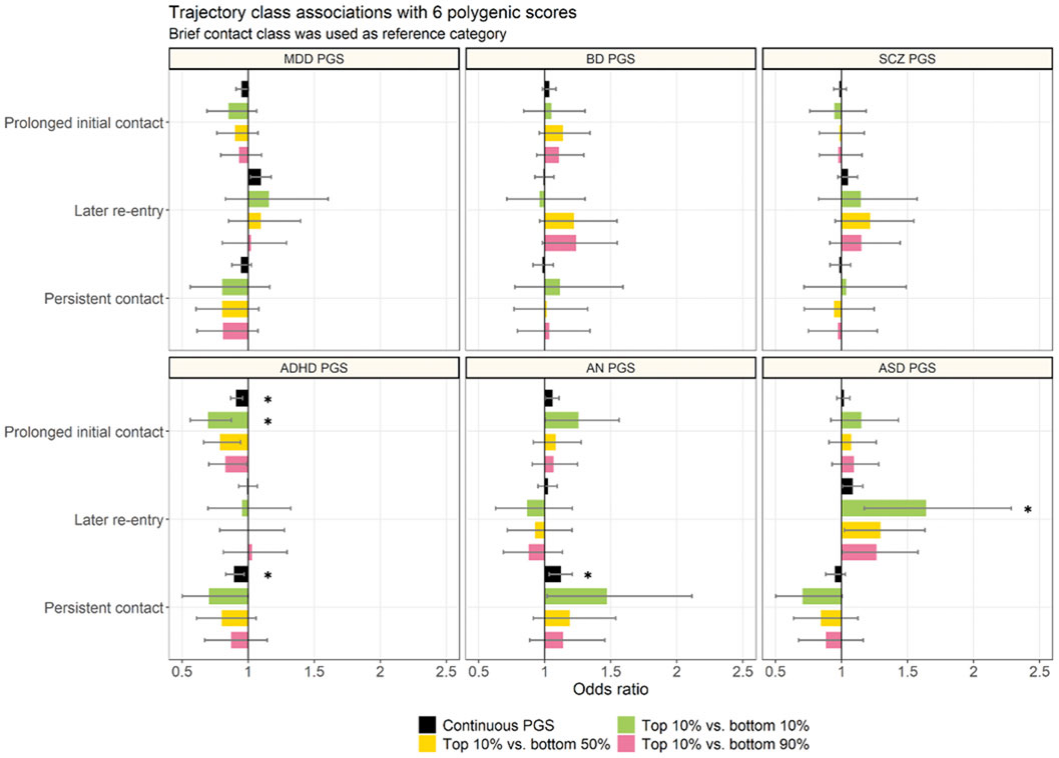
Associations between six polygenic scores (PGSs) and trajectories of hospital contacts for major depressive disorder (MDD). * significant at p < 0.0083 (Bonferroni-adjusted). MDD, major depressive disorder; ADHD, attention deficit hyperactivity disorder; ASD, autism spectrum disorder; BD, bipolar disorder; SCZ, schizophrenia; AN, anorexia nervosa. The brief contact class (class 1) was used as the reference category in the multinomial regressions.

Figure 2 shows the associations between the PGSs and trajectory class membership (full results in online Supplemental Tables 2–4). Relative to the *brief contact* class, higher PGS-MDD was associated with increased odds of membership in the *later re-entry* class (OR = 1.09 [95% CIs = 1.02–1.17], *p* = 0.01), but this result did not survive multiple testing. PGS-ADHD was associated with decreased odds of membership in the *prolonged initial contact* (OR = 0.91 [0.87–0.96], *p* = 0.0002) and *persistent contact* classes (OR = 0.90 [0.83–0.97], *p* = 0.007). PGS-AN was associated with increased odds of membership in the *persistent contact* class (OR = 1.12 [1.03–1.21], *p* = 0.005).

In analyses comparing the top decile of PGS to other groups, the negative association between PGS-ADHD and the *prolonged initial contact* class became stronger. For instance, compared to the bottom 10% of the PGS-ADHD distribution, the top 10% had an odds ratio of 0.70 [0.56–0.87] (*p* = 0.002). Additionally, there was a significant difference between the top vs bottom 10% of the PGS-ASD distribution with regards to membership in the *later re-entry* class (OR = 1.64 [1.17–2.29], *p* = 0.004).

Results from a multivariable model including all PGS variables are shown in online Supplemental Table 5. Overall, the estimates were highly similar to those obtained from models where the effect of each PGS was estimated separately. However, the association between PGS-ADHD and the *persistent contact* class no longer survived multiple testing (OR = 0.91 [0.84–0.99], *p* = 0.03).

### PGS and clinical course in the brief contact class

Of those with *brief contact*, 73.8% continued antidepressant medication treatment for MDD in primary care after exiting secondary care. Considering only prescriptions redeemed at least 6 months or 1 year after final discharge, the proportion decreased to 64.4% and 58.9%, respectively. Around half (49.7%) of the *brief contact* class re-entered the hospital system with a different psychiatric diagnosis (online Supplemental Table 6)**.** The most common diagnoses were anxiety disorders (26.6%), followed by personality disorders (18.9%), SCZ spectrum disorders (10.4%), substance abuse (8.0%), and childhood/adolescent-onset disorders (8.1%). A total of 1138 (16.6%) members of the *brief contact* class neither received treatment for another psychiatric diagnosis in secondary care or continued MDD treatment in primary care during the remaining follow-up. Considering only prescriptions redeemed at least 6 months or 1 year after final discharge, the number rose to 1540 (22.4%) and 1761 (25.6%), respectively.

**Figure 3.**
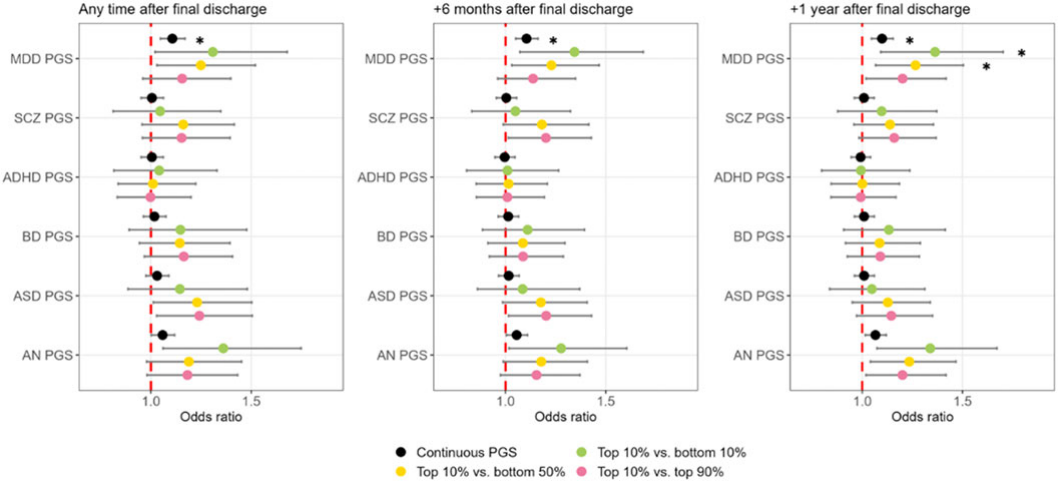
Associations between six polygenic scores (PGSs) and continued treatment in primary care in the remaining follow-up in individuals in the brief contact class. * = significant at p < 0.0083 (Bonferroni-adjusted). MDD, major depressive disorder; ADHD, attention deficit hyperactivity disorder; ASD, autism spectrum disorder; BD, bipolar disorder; SCZ, schizophrenia; AN, anorexia nervosa. Continued treatment in primary care was indexed by redeeming a prescription of antidepressants in the remaining follow-up period after the final discharge from a psychiatric hospital for MDD. Three analyses were conducted: (1) including prescriptions redeemed any time after final discharge, (2) including prescriptions redeemed only 6 months or more after final discharge, and (3) including prescriptions redeemed only 12 months or more after final discharge.

Higher PGS-MDD was significantly associated with further medical treatment for MDD in primary care (OR = 1.11 [1.05–1.17], *p* = 0.0003) (Fig. 3, full results in online Supplemental Tables 7–10). This effect was similar when considering prescriptions redeemed after 6 months and 1 year after the final discharge date, and in the mutually adjusted models (online Supplemental Table 8). The effect was particularly apparent for individuals in the top decile of PGS for MDD (relative to the bottom 10%: OR = 1.36 [1.09–1.70], *p* = 0.006) (online Supplemental Table 9).

**Figure 4.**
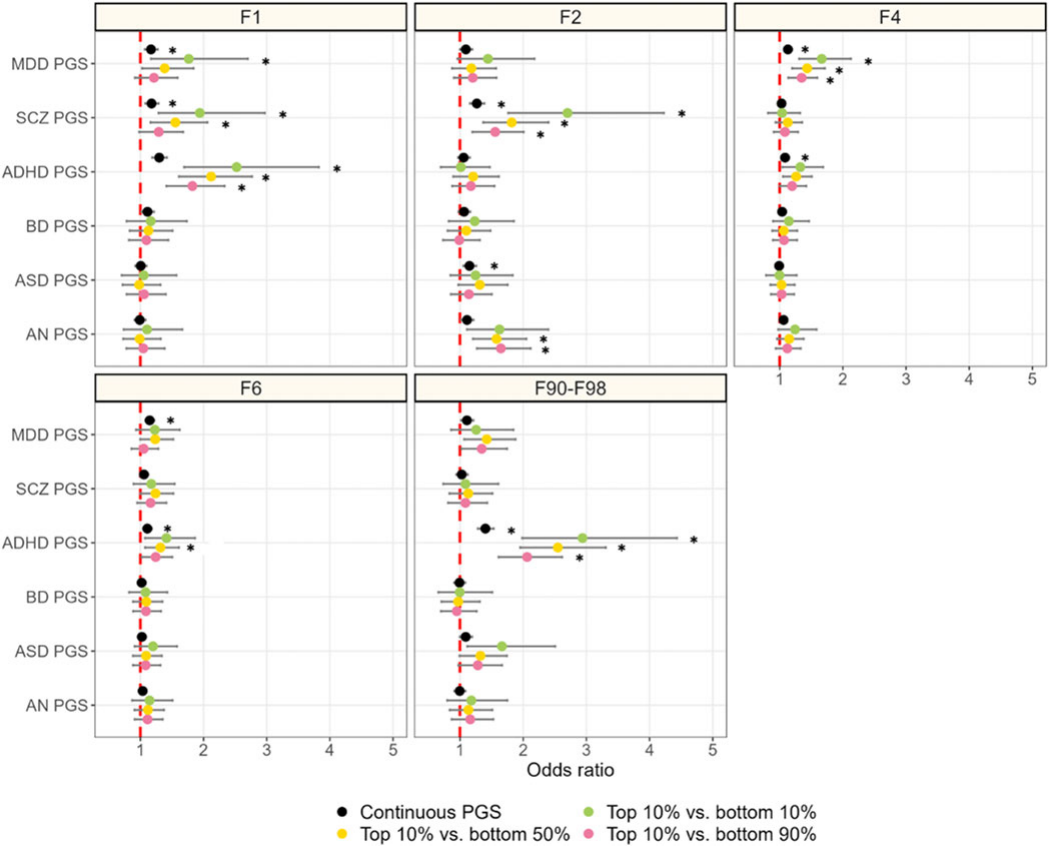
Associations between polygenic scores (PGSs) and treatment for other psychiatric disorders in secondary care in the remaining follow-up in individuals in the brief contact class. * = significant at p < 0.0083 (Bonferroni-adjusted). MDD, major depressive disorder; ADHD, attention deficit hyperactivity disorder; ASD. autism spectrum disorder; BD, bipolar disorder; SCZ, schizophrenia; AN, anorexia nervosa. F categories refer to broad psychiatric diagnostic categories in the 10th edition of the International Classification of Diseases (ICD-10). • ICD-10 F1 disorders – Mental and behavioural disorder due to psychoative substance use • ICD-10 F2 disorders – Schizophrenia, schizotypal and delusional disorder • ICD-10 F4 disorders – Neurotic, stress-related and somatoform disorders • ICD-10 F6 disorders – Personality disorders • ICD-10 F90-F98 disorders – Behavioural and emotional disorders with onset usually occurring in childhood and adolescence

Associations between PGSs and subsequent secondary care for other psychiatric disorders are shown in Fig. 4 (full results in online Supplemental Tables 11–13). PGS-MDD (OR = 1.17, [1.07–1.28], 0.0006), PGS-ADHD (OR = 1.30 [1.19–1.42], *p* < 0.0001), and PGS-SCZ (OR = 1.18 [1.08–1.29], *p* = 0.0003) were associated with subsequent secondary care for substance abuse. PGS-SCZ (OR = 1.27 [1.16–1.38], *p* < 0.0001) and PGS-ASD (OR = 1.15 [1.05–1.26], *p* = 0.002) were associated with subsequent SCZ spectrum disorders. PGS-MDD (OR = 1.13 [1.07–1.19], *p* < 0.0001) and PGS-ADHD (OR = 1.08 [1.03–1.14], *p* = 0.003) were associated with anxiety disorders. PGS-MDD (OR = 1.15 [1.08–1.22], *p* < 0.0001) and PGS-ADHD (OR = 1.11 [1.04–1.18], *p* = 0.0008) were associated with personality disorders. Finally, PGS-ADHD (OR = 1.40 [1.28–1.53], *p* < 0.0001) was associated with childhood/adolescent-onset disorders. In the multivariable models **(**online Supplemental Table 14**),** only PGS-ADHD was independently associated with subsequent treatment for substance abuse (OR = 1.30, [1.18–1.43], *p* < 0.0001) and child/adolescent-onset disorders (OR = 1.42, [1.28–1.55], *p* = <0.0001), only PGS-SCZ was independently associated with subsequent treatment for SCZ spectrum disorders (OR = 1.26, [1.14–1.40], *p* = <0.0001), and only PGS-MDD was independently associated with subsequent treatment for anxiety (OR = 1.11, [1.05–1.18], *p* = 0.0003) and personality (OR = 1.13, [1.06–1.20], *p* = 0.0004) disorders.

When comparing the top decile of the PGS distribution to the bottom 10%, 50%, and 90%, the most notable findings were that, compared to the continuous variable, the top decile for PGS-ADHD showed a large increase in effect size for substance abuse (e.g. top 10% vs bottom 10%: OR = 2.52 [1.69–3.82], *p* < 0.0001). Similarly, the top decile of SCZ-PGS showed a large increase in effect size for SCZ spectrum disorders (e.g. top 10% vs bottom 10%: OR = 2.70 [1.76–4.22], *p* < 0.0001). Last, the top decile of PGS-ADHD showed an increase in effect size for child/adolescent-onset disorders, especially for the bottom 10% vs top 10% (OR = 2.94 [1.98–4.43], *p* < 0.0001) and vs bottom 50% (OR = 2.55 [1.95–3.31], *p* < 0.0001).

### Parental history

Parental history of MDD was associated with membership in the *later re-entry* class (OR = 1.31 [1.09–1.57], *p* = 0.003) (online Supplemental Table 15 and online Supplemental Figure 5), and with subsequent antidepressant treatment in the *brief contact* class any time (OR = 1.21 [1.04–1.41], *p* = 0.016) and 6 + months after final discharge from secondary care (OR = 1.24 [1.08–1.43], *p* = 0.002) (online Supplemental Table 16 and online Supplemental Figure 6).

Parental history of psychiatric disorders was also associated with transitioning to other psychiatric diagnoses among individuals in the *brief contact* class (online Supplemental Table 17 and online Supplemental Figure 7). Notably, having a parental history of SCZ spectrum disorders was associated with returning to secondary care with a diagnosis of SCZ spectrum disorder (OR = 1.65 [1.14–2.32], *p* = 0.005) or anxiety disorders (OR = 1.50 [1.15–1.96], *p* = 0.003). Additionally, parental history of BD (OR = 1.83 [1.20–2.71], *p* = 0.004) was significantly associated with childhood/adolescent-onset disorders.

### Age-at-index episode

Age-stratified models showed that the nominal association between PGS-MDD and *later re-entry* was primarily driven by individuals ages 21–25 at first diagnosis in secondary care (OR = 1.16 [1.02–1.32], *p* = 0.02). Although the effect size in this group was almost double that in the full cohort, this result was still not significant at the Bonferroni-corrected level, possibly due to reduced statistical power. In contrast, the negative association between PGS-ADHD and the *persistent contact* class was driven primarily by individuals 10–20 years old at first diagnosis (online Supplemental Table 18 and online Supplemental Figure 3). Likewise, the positive association between PGS-AN and the *persistent contact* class was driven by those diagnosed at younger ages (10–20 years). The association between PGS-ADHD and subsequent secondary treatment for childhood/adolescent-onset disorders in the *brief contact* class was similar across age groups (online Supplemental Table 19 and online Supplemental Figure 4).

## Discussion

The goal of this study was to investigate associations between polygenic liabilities for six major psychiatric disorders and 7-year treatment trajectory patterns in individuals diagnosed with early-onset MDD in secondary care. As in our prior study (Musliner et al., 2016) we identified four patterns of hospital-based contacts for MDD: the most common pattern (65%) was characterized by *brief contact* while the remaining patients showed course trajectories characterized by *prolonged initial contact* (20%), *later re-entry* (8%), *and persistent contact* (7%). Among individuals in the *brief contact* class, we found that most (75%) continued medication for MDD in primary care and just under half received treatment for a different psychiatric disorder in secondary care. However, a portion of this group (17–26%) neither continued treatment with antidepressants nor converted to a different diagnosis. Some of these individuals likely recovered from their MDD episode and did not experience another one during the study period. Prior research (Eaton et al., 2008) has found that up to 50% of individuals with MDD experience only a single episode. However, some may have chosen to pursue treatment through a private practicing psychiatrist or psychologist. In addition, social, economic, and cultural pressures could have hindered access to continued treatment. Therefore, we cannot be certain that these patients recovered, only that they were no longer actively engaged with the public healthcare sector for MDD.

We found that higher genetic liability for MDD was associated with membership in the *later-re-entry* class relative to the *brief contact* class. The association between PGS-MDD and the later re-entry class was suggestive but did not survive multiple testing either overall, or in the age-stratified analyses, where it appeared to be driven by individuals 21–25 years at first MDD diagnosis. However, parental history of MDD was significantly associated with the later re-entry trajectory, which, along with the suggestive results for PGS-MDD, aligns with evidence that individuals with a higher genetic liability for MDD are more likely to experience recurrent than single-episode MDD (Als et al., 2023; Mitchell et al., 2022; Musliner et al., 2021; Wray et al., 2018). It is possible that the *later re-entry* patients represent a more canonical group of recurrent depression cases, while patients in the other classes represent more complex clinical presentations. PGS-MDD and parental history of MDD were also positively associated with continued medication treatment in primary care among those with *brief contact*, suggesting continued symptoms and/or, potentially, treatment responsiveness, which warrants further study. However, it should be noted that the effect sizes were small, even for those with the highest PGS-MDD scores. Therefore, PGS-MDD is unlikely to be of use for predicting admission trajectories in clinical settings.

Several PGSs for other psychiatric disorders were also associated with treatment trajectories. The strongest results were for PGS-ADHD, which was associated with increased likelihood of a *brief contact* pattern relative to the *prolonged initial contact* and *persistent contact* patterns, particularly for younger individuals. Once leaving secondary care, those with high PGS-ADHD were also 2–3 times as likely to return with a different diagnosis, particularly child/adolescent-onset disorders, but also substance abuse and personality disorders. It is possible that these individuals represent early-onset MDD cases who have been misdiagnosed or whose depressive symptoms occur within the context of comorbidities. Perhaps PGS-ADHD could even be a useful addition to predictive algorithms designed to aid in differential diagnosis, however further research is needed to investigate that possibility. We also found an association between PGS-AN and the *persistent contact* class which was statistically significant and unchanged in the mutually adjusted model, suggesting that this genetic liability is associated with longer periods of MDD treatment in secondary care.

## Limitations

This study has several limitations. First, administrative records of hospital contacts for psychiatric disorders are indirect measures of actual illness and thus do not translate perfectly to depressive episodes. Furthermore, contact with secondary care is influenced by other factors such as treatment seeking or referral policies which could influence the associations. Second, our results may not generalize to individuals with MDD who (a) do not receive treatment and (b) are treated solely in primary care. However, the Danish system mandates psychiatric oversight of all medical treatment for individuals under 25 (Ministerium: Indenrigs- og Sundhedsministeriet, 2014). Therefore, our sample may be more representative than studies of older-onset cases. Third, inclusion in the sample required survival for at least seven years after initial diagnosis. The removal of individuals who died during this period could have biased results, although this is unlikely as death was rare (*N* = 176, 0.006% of original sample). Fourth, although the internally-derived portions of the PGS variables were all derived from iPSYCH, the externally-derived portions were based on results from discovery datasets with varying phenotyping methods and sample size. Thus, some of the scores were more statistically powered than others, which should be taken into consideration when interpreting the results. Last, our analyses were conducted on individuals of European ancestry and may thus not generalize to other populations. Psychiatric genetics researchers are currently grappling with the challenge of addressing the disproportionate number of studies published on Europeans. We were restricted to conducting such analyses due to the demographic make-up of the iPSYCH sample. However, the limitation is worth mentioning to draw attention to the potential widening of health inequalities unless we, as a research community, actively push for greater emphasis on underrepresented populations.

## Conclusions

Among patients with early-onset MDD, we found small associations between polygenic liabilities for psychiatric disorders and treatment trajectories in primary and secondary care. These results provide some insight into how individual differences in genetic risk, for MDD and other psychiatric disorders, are related to differences in treatment trajectory, however, the effect sizes were modest and thus the associations are unlikely to be useful for prospective prediction of clinical course.

## Supporting information

Mundy et al. supplementary material Mundy et al. supplementary material

## Supplementary material

The supplementary material for this article can be found at https://doi.org/10.1017/S0033291724002186.
