# Supplementary material for: Polygenic liabilities and treatment trajectories in early-onset depression: a Danish register-based study
Source: Psychol Med. 2024 Oct 14;54(14):3828–37. doi: 10.1017/S0033291724002186 (PMC11578915; doi:10.1017/S0033291724002186)
Supplement: Mundy et al. supplementary material [file S0033291724002186sup001.docx]

Supplemental Material

Contents

[Abbreviations 3](#_Toc171087949)

[STable 1. Fit statistics for Latent Class Growth Analysis (LCGA) models with 1-6 trajectory classes. 4](#_Toc171087950)

[STable 2. Associations between continuous polygenic scores (PGSs) and trajectory classes. 5](#_Toc171087951)

[STable 3. Associations between dichotomized (10%) polygenic scores (PGSs) and trajectory classes. 6](#_Toc171087952)

[STable 4. Associations between dichotomized (5%) polygenic scores (PGSs) and trajectory classes. 9](#_Toc171087953)

[STable 5. Mutually adjusted associations between PGS and trajectory class membership 12](#_Toc171087954)

[Stable 6. Frequency of treatment for other psychiatric diagnoses and continued treatment in primary care in the remaining follow-up in individuals in the brief contact class. 14](#_Toc171087955)

[STable 7. Associations between continuous polygenic scores (PGSs) and continued treatment in primary care in the remaining follow-up in individuals in the brief contact class. 16](#_Toc171087956)

[STable 8. Associations between continuous polygenic scores (PGSs) and continued treatment in primary care in the remaining follow-up in individuals in the brief contact class – mutually adjusted models. 17](#_Toc171087957)

[Stable 9. Associations between dichotomized (10%) polygenic scores (PGSs) and continued treatment in primary care in the remaining follow-up in individuals in the brief contact class. 19](#_Toc171087958)

[STable 10. Associations between dichotomized (5%) polygenic scores (PGSs) and continued treatment in primary care in the remaining follow-up in individuals in the brief contact class. 22](#_Toc171087959)

[STable 11. Association between continuous polygenic scores (PGSs) and treatment for other psychiatric disorders in secondary care in the remaining follow-up in individuals in the brief contact class. 25](#_Toc171087960)

[STable 12. Association between dichotomized (10%) polygenic scores (PGSs) and treatment for other psychiatric disorders in secondary care in the remaining follow-up in individuals in the brief contact class. 27](#_Toc171087961)

[STable 13. Association between dichotomized (5%) polygenic scores (PGSs) and treatment for other psychiatric disorders in secondary care in the remaining follow-up in individuals in the brief contact class. 32](#_Toc171087962)

[STable 14. Association between continuous polygenic scores (PGSs) and treatment for other psychiatric disorders in secondary care in the remaining follow-up in individuals in the brief contact class – mutually adjusted models. 37](#_Toc171087963)

[Stable 15. Associations between parental history of psychiatric disorders and trajectory class membership. 39](#_Toc171087964)

[STable 16. Associations between parental history of psychiatric disorders and continued treatment in primary care for MDD in the remaining follow-up in individuals in the brief contact class. 40](#_Toc171087965)

[STable 17. Associations between parental history of psychiatric disorders and treatment for other psychiatric disorders in secondary care in the remaining follow-up in individuals in the brief contact class. 41](#_Toc171087966)

[STable 18. Associations between continuous polygenic scores (PGSs) and trajectory classes stratified by age-at-index episode. 43](#_Toc171087967)

[STable 19. Associations between continuous polygenic scores (PGSs) and treatment for other psychiatric disorders in secondary care in the remaining follow-up in individuals in the brief contact class stratified by age-at-index episode. 46](#_Toc171087968)

[SFigure 1. Sample selection process 50](#_Toc171087969)

[SFigure 2. Trajectory patterns for Latent Class Growth Analysis (LCGA) models with 1-6 classes. 51](#_Toc171087970)

[SFigure 3. Associations between continuous polygenic scores (PGSs) and trajectory classes stratified by age-at-index episode. 52](#_Toc171087971)

[SFigure 4. Associations between continuous polygenic scores (PGSs) and treatment for other psychiatric diagnoses in secondary care in the remaining follow-up in individuals the brief contact class stratified by age-at-index episode. 53](#_Toc171087972)

[SFigure 5. Associations between parental history of psychiatric disorders and trajectory class membership. 54](#_Toc171087973)

[SFigure 6. Associations between parental history of psychiatric disorders and continued treatment in primary care in the remaining follow-up in individuals in the brief contact class. 55](#_Toc171087974)

[SFigure 7. Associations between parental history of psychiatric disorders and treatment for other psychiatric disorders in secondary care in the remaining follow-up in individuals in the brief contact class. 56](#_Toc171087975)

# Abbreviations

*ADHD= attention deficit hyperactivity disorder*

*AN=Anorexia nervosa*

*ASD=autism spectrum disorder*

*BD=bipolar disorder*

*CI=confidence intervals*

*MDD=major depressive disorder*

*SCZ=schizophrenia*

*All confidence intervals (CIs) refer to 95% CIs.*

# STable 1. Fit statistics for Latent Class Growth Analysis (LCGA) models with 1-6 trajectory classes.

| Model | BIC | AIC | Class 1 | Class 2 | Class 3 | Class 4 | Class 5 | Class 6 |
| --- | --- | --- | --- | --- | --- | --- | --- | --- |
| 1 Class Model | -47723.15 | -47708.62 | 100% | . | . | . | . | . |
| 2 Class Model | -38097.80 | -38065.10 | 97% | 96% | . | . | . | . |
| 3 Class Model | -35393.85 | -35342.98 | 98% | 86% | 94% | . | . | . |
| 4 Class Model | -34027.89 | -33958.86 | 98% | 90% | 92% | 94% | . | . |
| 5 Class Model | -33282.81 | -33195.61 | 95% | 95% | 93% | 91% | 93% | . |
| 6 Class Model | -32694.18 | -32588.82 | 96% | 87% | 94% | 93% | 94% | 94% |

# STable 2. Associations between continuous polygenic scores (PGSs) and trajectory classes.

| Trajectory class | PGS | OR | SE | p-value | Lower CI | Upper CI |
| --- | --- | --- | --- | --- | --- | --- |
| Prolonged initial contact | PGS-MDD | 0.96 | 0.02 | 6.60x10-02 | 0.91 | 1.00 |
| Later re-entry | PGS-MDD | 1.09 | 0.04 | 1.47x10-02 | 1.02 | 1.17 |
| Persistent contact | PGS-MDD | 0.95 | 0.04 | 1.89x10-01 | 0.88 | 1.03 |
| Prolonged initial contact | PGS-ADHD | 0.91 | 0.02 | 2.12x10-04 | 0.87 | 0.96 |
| Later re-entry | PGS-ADHD | 1.00 | 0.04 | 9.32x10-01 | 0.93 | 1.07 |
| Persistent contact | PGS-ADHD | 0.90 | 0.04 | 6.99x10-03 | 0.83 | 0.97 |
| Prolonged initial contact | PGS-ASD | 1.01 | 0.02 | 5.79x10-01 | 0.97 | 1.06 |
| Later re-entry | PGS-ASD | 1.08 | 0.04 | 3.15x10-02 | 1.01 | 1.16 |
| Persistent contact | PGS-ASD | 0.95 | 0.04 | 2.20x10-01 | 0.88 | 1.03 |
| Prolonged initial contact | PGS-BD | 1.03 | 0.02 | 2.00x10-01 | 0.98 | 1.08 |
| Later re-entry | PGS-BD | 0.99 | 0.04 | 8.68x10-01 | 0.92 | 1.07 |
| Persistent contact | PGS-BD | 0.99 | 0.04 | 7.15x10-01 | 0.91 | 1.07 |
| Prolonged initial contact | PGS-SCZ | 0.99 | 0.02 | 6.37x10-01 | 0.94 | 1.04 |
| Later re-entry | PGS-SCZ | 1.05 | 0.04 | 2.31x10-01 | 0.97 | 1.12 |
| Persistent contact | PGS-SCZ | 0.99 | 0.04 | 7.83x10-01 | 0.91 | 1.07 |
| Prolonged initial contact | PGS-AN | 1.06 | 0.02 | 3.06x10-02 | 1.01 | 1.11 |
| Later re-entry | PGS-AN | 1.02 | 0.04 | 5.99x10-01 | 0.95 | 1.10 |
| Persistent contact | PGS-AN | 1.12 | 0.04 | 5.00x10-03 | 1.03 | 1.21 |

*The brief contact class (class 1) was used as the reference category in multinomial regressions. Dark grey background represents results significant at the Bonferroni-adjusted alpha level of 0.00083.*

# STable 3. Associations between dichotomized (10%) polygenic scores (PGSs) and trajectory classes.

| Trajectory class | PGS | OR | SE | p-value | Lower CI | Upper CI |
| --- | --- | --- | --- | --- | --- | --- |
| Prolonged initial contact | PGS-MDD top 10% vs. bottom 10% | 0.86 | 0.11 | 1.63x10-01 | 0.69 | 1.06 |
| Later re-entry | PGS-MDD top 10% vs. bottom 10% | 1.15 | 0.17 | 3.98x10-01 | 0.83 | 1.61 |
| Persistent contact | PGS-MDD top 10% vs. bottom 10% | 0.81 | 0.18 | 2.52x10-01 | 0.56 | 1.16 |
| Prolonged initial contact | PGS-ADHD top 10% vs. bottom 10% | 0.70 | 0.11 | 1.60x10-03 | 0.56 | 0.87 |
| Later re-entry | PGS-ADHD top 10% vs. bottom 10% | 0.96 | 0.16 | 7.99x10-01 | 0.70 | 1.32 |
| Persistent contact | PGS-ADHD top 10% vs. bottom 10% | 0.71 | 0.18 | 5.17x10-02 | 0.50 | 1.00 |
| Prolonged initial contact | PGS-SCZ top 10% vs. bottom 10% | 0.95 | 0.11 | 6.54x10-01 | 0.76 | 1.19 |
| Later re-entry | PGS-SCZ top 10% vs. bottom 10% | 1.14 | 0.16 | 4.19x10-01 | 0.83 | 1.57 |
| Persistent contact | PGS-SCZ top 10% vs. bottom 10% | 1.03 | 0.19 | 8.57x10-01 | 0.72 | 1.49 |
| Prolonged initial contact | PGS-BD top 10% vs. bottom 10% | 1.05 | 0.11 | 6.74x10-01 | 0.84 | 1.31 |
| Later re-entry | PGS-BD top 10% vs. bottom 10% | 0.96 | 0.15 | 8.17x10-01 | 0.71 | 1.31 |
| Persistent contact | PGS-BD top 10% vs. bottom 10% | 1.11 | 0.18 | 5.68x10-01 | 0.77 | 1.59 |
| Prolonged initial contact | PGS-AN top 10% vs. bottom 10% | 1.25 | 0.11 | 4.75x10-02 | 1.00 | 1.56 |
| Later re-entry | PGS-AN top 10% vs. bottom 10% | 0.87 | 0.17 | 4.08x10-01 | 0.63 | 1.21 |
| Persistent contact | PGS-AN top 10% vs. bottom 10% | 1.47 | 0.19 | 3.98x10-02 | 1.02 | 2.11 |
| Prolonged initial contact | PGS-ASD top 10% vs. bottom 10% | 1.15 | 0.11 | 2.21x10-01 | 0.92 | 1.43 |
| Later re-entry | PGS-ASD top 10% vs. bottom 10% | 1.64 | 0.17 | 3.63x10-03 | 1.17 | 2.29 |
| Persistent contact | PGS-ASD top 10% vs. bottom 10% | 0.71 | 0.18 | 5.26x10-02 | 0.50 | 1.00 |
| Prolonged initial contact | PGS-MDD top 10% vs. bottom 90% | 0.93 | 0.08 | 4.23x10-01 | 0.79 | 1.10 |
| Later re-entry | PGS-MDD top 10% vs. bottom 90% | 1.02 | 0.12 | 8.76x10-01 | 0.80 | 1.29 |
| Persistent contact | PGS-MDD top 10% vs. bottom 90% | 0.81 | 0.14 | 1.45x10-01 | 0.61 | 1.07 |
| Prolonged initial contact | PGS-ADHD top 10% vs. bottom 90% | 0.83 | 0.09 | 3.49x10-02 | 0.70 | 0.99 |
| Later re-entry | PGS-ADHD top 10% vs. bottom 90% | 1.03 | 0.12 | 8.31x10-01 | 0.81 | 1.29 |
| Persistent contact | PGS-ADHD top 10% vs. bottom 90% | 0.88 | 0.14 | 3.35x10-01 | 0.67 | 1.15 |
| Prolonged initial contact | PGS-SCZ top 10% vs. bottom 90% | 0.98 | 0.08 | 8.22x10-01 | 0.83 | 1.16 |
| Later re-entry | PGS-SCZ top 10% vs. bottom 90% | 1.15 | 0.12 | 2.38x10-01 | 0.91 | 1.45 |
| Persistent contact | PGS-SCZ top 10% vs. bottom 90% | 0.98 | 0.13 | 8.65x10-01 | 0.75 | 1.27 |
| Prolonged initial contact | PGS-BD top 10% vs. bottom 90% | 1.10 | 0.08 | 2.36x10-01 | 0.94 | 1.29 |
| Later re-entry | PGS-BD top 10% vs. bottom 90% | 1.23 | 0.12 | 7.02x10-02 | 0.98 | 1.55 |
| Persistent contact | PGS-BD top 10% vs. bottom 90% | 1.03 | 0.13 | 8.14x10-01 | 0.79 | 1.34 |
| Prolonged initial contact | PGS-AN top 10% vs. bottom 90% | 1.06 | 0.08 | 4.51x10-01 | 0.91 | 1.25 |
| Later re-entry | PGS-AN top 10% vs. bottom 90% | 0.88 | 0.13 | 3.28x10-01 | 0.69 | 1.13 |
| Persistent contact | PGS-AN top 10% vs. bottom 90% | 1.14 | 0.13 | 3.18x10-01 | 0.89 | 1.46 |
| Prolonged initial contact | PGS-ASD top 10% vs. bottom 90% | 1.09 | 0.08 | 2.81x10-01 | 0.93 | 1.28 |
| Later re-entry | PGS-ASD top 10% vs. bottom 90% | 1.26 | 0.11 | 4.13x10-02 | 1.01 | 1.58 |
| Persistent contact | PGS-ASD top 10% vs. bottom 90% | 0.89 | 0.14 | 3.89x10-01 | 0.68 | 1.17 |
| Prolonged initial contact | PGS-MDD top 10% vs. bottom 50% | 0.91 | 0.09 | 2.55x10-01 | 0.76 | 1.07 |
| Later re-entry | PGS-MDD top 10% vs. bottom 50% | 1.09 | 0.13 | 4.88x10-01 | 0.85 | 1.40 |
| Persistent contact | PGS-MDD top 10% vs. bottom 50% | 0.81 | 0.15 | 1.52x10-01 | 0.61 | 1.08 |
| Prolonged initial contact | PGS-ADHD top 10% vs. bottom 50% | 0.79 | 0.09 | 8.95x10-03 | 0.66 | 0.94 |
| Later re-entry | PGS-ADHD top 10% vs. bottom 50% | 1.00 | 0.12 | 9.99x10-01 | 0.78 | 1.28 |
| Persistent contact | PGS-ADHD top 10% vs. bottom 50% | 0.80 | 0.14 | 1.22x10-01 | 0.61 | 1.06 |
| Prolonged initial contact | PGS-SCZ top 10% vs. bottom 50% | 0.99 | 0.09 | 9.02x10-01 | 0.83 | 1.17 |
| Later re-entry | PGS-SCZ top 10% vs. bottom 50% | 1.21 | 0.12 | 1.16x10-01 | 0.95 | 1.55 |
| Persistent contact | PGS-SCZ top 10% vs. bottom 50% | 0.95 | 0.14 | 7.02x10-01 | 0.72 | 1.25 |
| Prolonged initial contact | PGS-BD top 10% vs. bottom 50% | 1.13 | 0.09 | 1.42x10-01 | 0.96 | 1.34 |
| Later re-entry | PGS-BD top 10% vs. bottom 50% | 1.22 | 0.12 | 1.02x10-01 | 0.96 | 1.55 |
| Persistent contact | PGS-BD top 10% vs. bottom 50% | 1.01 | 0.14 | 9.47x10-01 | 0.77 | 1.33 |
| Prolonged initial contact | PGS-AN top 10% vs. bottom 50% | 1.08 | 0.09 | 3.74x10-01 | 0.91 | 1.27 |
| Later re-entry | PGS-AN top 10% vs. bottom 50% | 0.93 | 0.13 | 5.80x10-01 | 0.72 | 1.21 |
| Persistent contact | PGS-AN top 10% vs. bottom 50% | 1.19 | 0.13 | 2.00x10-01 | 0.91 | 1.54 |
| Prolonged initial contact | PGS-ASD top 10% vs. bottom 50% | 1.07 | 0.09 | 4.22x10-01 | 0.91 | 1.26 |
| Later re-entry | PGS-ASD top 10% vs. bottom 50% | 1.29 | 0.12 | 3.25x10-02 | 1.02 | 1.63 |
| Persistent contact | PGS-ASD top 10% vs. bottom 50% | 0.85 | 0.14 | 2.51x10-01 | 0.64 | 1.12 |

*The brief contact class (class 1) was used as the reference category in multinomial regressions. Dark grey background represents results significant at the Bonferroni-adjusted alpha level of 0.00083.*

# STable 4. Associations between dichotomized (5%) polygenic scores (PGSs) and trajectory classes.

| Trajectory class | PGS | OR | SE | p-value | Lower CI | Upper CI |
| --- | --- | --- | --- | --- | --- | --- |
| Prolonged initial contact | PGS-MDD top 5% vs. bottom 5% | 0.86 | 0.16 | 3.33x10-01 | 0.63 | 1.17 |
| Later re-entry | PGS-MDD top 5% vs. bottom 5% | 1.38 | 0.23 | 1.65x10-01 | 0.88 | 2.18 |
| Persistent contact | PGS-MDD top 5% vs. bottom 5% | 0.74 | 0.29 | 3.06x10-01 | 0.42 | 1.31 |
| Prolonged initial contact | PGS-ADHD top 5% vs. bottom 5% | 0.70 | 0.16 | 2.80x10-02 | 0.51 | 0.96 |
| Later re-entry | PGS-ADHD top 5% vs. bottom 5% | 0.94 | 0.22 | 7.67x10-01 | 0.61 | 1.44 |
| Persistent contact | PGS-ADHD top 5% vs. bottom 5% | 1.04 | 0.26 | 8.77x10-01 | 0.62 | 1.74 |
| Prolonged initial contact | PGS-SCZ top 5% vs. bottom 5% | 0.96 | 0.16 | 7.97x10-01 | 0.70 | 1.32 |
| Later re-entry | PGS-SCZ top 5% vs. bottom 5% | 1.08 | 0.23 | 7.43x10-01 | 0.68 | 1.71 |
| Persistent contact | PGS-SCZ top 5% vs. bottom 5% | 1.01 | 0.27 | 9.58x10-01 | 0.60 | 1.72 |
| Prolonged initial contact | PGS-BD top 5% vs. bottom 5% | 1.30 | 0.16 | 9.87x10-02 | 0.95 | 1.78 |
| Later re-entry | PGS-BD top 5% vs. bottom 5% | 0.93 | 0.22 | 7.57x10-01 | 0.60 | 1.45 |
| Persistent contact | PGS-BD top 5% vs. bottom 5% | 1.33 | 0.27 | 2.94x10-01 | 0.78 | 2.25 |
| Prolonged initial contact | PGS-AN top 5% vs. bottom 5% | 1.58 | 0.16 | 5.25x10-03 | 1.15 | 2.19 |
| Later re-entry | PGS-AN top 5% vs. bottom 5% | 0.98 | 0.23 | 9.15x10-01 | 0.62 | 1.53 |
| Persistent contact | PGS-AN top 5% vs. bottom 5% | 1.39 | 0.27 | 2.21x10-01 | 0.82 | 2.37 |
| Prolonged initial contact | PGS-ASD top 5% vs. bottom 5% | 1.34 | 0.16 | 6.80x10-02 | 0.98 | 1.82 |
| Later re-entry | PGS-ASD top 5% vs. bottom 5% | 1.66 | 0.24 | 3.84x10-02 | 1.03 | 2.67 |
| Persistent contact | PGS-ASD top 5% vs. bottom 5% | 0.89 | 0.25 | 6.48x10-01 | 0.55 | 1.45 |
| Prolonged initial contact | PGS-MDD top 5% vs. bottom 95% | 0.93 | 0.12 | 5.58x10-01 | 0.74 | 1.17 |
| Later re-entry | PGS-MDD top 5% vs. bottom 95% | 1.19 | 0.16 | 2.75x10-01 | 0.87 | 1.61 |
| Persistent contact | PGS-MDD top 5% vs. bottom 95% | 0.64 | 0.22 | 4.05x10-02 | 0.41 | 0.98 |
| Prolonged initial contact | PGS-ADHD top 5% vs. bottom 95% | 0.82 | 0.12 | 1.04x10-01 | 0.65 | 1.04 |
| Later re-entry | PGS-ADHD top 5% vs. bottom 95% | 1.20 | 0.15 | 2.48x10-01 | 0.88 | 1.62 |
| Persistent contact | PGS-ADHD top 5% vs. bottom 95% | 0.97 | 0.18 | 8.82x10-01 | 0.68 | 1.39 |
| Prolonged initial contact | PGS-SCZ top 5% vs. bottom 95% | 0.98 | 0.12 | 8.93x10-01 | 0.79 | 1.23 |
| Later re-entry | PGS-SCZ top 5% vs. bottom 95% | 1.12 | 0.16 | 4.99x10-01 | 0.81 | 1.54 |
| Persistent contact | PGS-SCZ top 5% vs. bottom 95% | 1.00 | 0.18 | 9.96x10-01 | 0.70 | 1.43 |
| Prolonged initial contact | PGS-BD top 5% vs. bottom 95% | 1.24 | 0.11 | 4.94x10-02 | 1.00 | 1.54 |
| Later re-entry | PGS-BD top 5% vs. bottom 95% | 1.15 | 0.16 | 3.95x10-01 | 0.83 | 1.59 |
| Persistent contact | PGS-BD top 5% vs. bottom 95% | 1.04 | 0.19 | 8.29x10-01 | 0.72 | 1.50 |
| Prolonged initial contact | PGS-AN top 5% vs. bottom 95% | 1.13 | 0.11 | 2.70x10-01 | 0.91 | 1.40 |
| Later re-entry | PGS-AN top 5% vs. bottom 95% | 1.05 | 0.17 | 7.83x10-01 | 0.76 | 1.45 |
| Persistent contact | PGS-AN top 5% vs. bottom 95% | 1.00 | 0.18 | 9.80x10-01 | 0.70 | 1.43 |
| Prolonged initial contact | PGS-ASD top 5% vs. bottom 95% | 1.20 | 0.11 | 1.03x10-01 | 0.96 | 1.49 |
| Later re-entry | PGS-ASD top 5% vs. bottom 95% | 1.20 | 0.16 | 2.53x10-01 | 0.88 | 1.64 |
| Persistent contact | PGS-ASD top 5% vs. bottom 95% | 1.04 | 0.18 | 8.42x10-01 | 0.72 | 1.49 |
| Prolonged initial contact | PGS-MDD top 5% vs. bottom 50% | 0.90 | 0.12 | 3.92x10-01 | 0.72 | 1.14 |
| Later re-entry | PGS-MDD top 5% vs. bottom 50% | 1.26 | 0.16 | 1.50x10-01 | 0.92 | 1.73 |
| Persistent contact | PGS-MDD top 5% vs. bottom 50% | 0.64 | 0.22 | 4.42x10-02 | 0.41 | 0.99 |
| Prolonged initial contact | PGS-ADHD top 5% vs. bottom 50% | 0.77 | 0.12 | 3.80x10-02 | 0.61 | 0.99 |
| Later re-entry | PGS-ADHD top 5% vs. bottom 50% | 1.16 | 0.16 | 3.55x10-01 | 0.85 | 1.58 |
| Persistent contact | PGS-ADHD top 5% vs. bottom 50% | 0.88 | 0.19 | 5.12x10-01 | 0.61 | 1.28 |
| Prolonged initial contact | PGS-SCZ top 5% vs. bottom 50% | 0.99 | 0.12 | 9.41x10-01 | 0.79 | 1.25 |
| Later re-entry | PGS-SCZ top 5% vs. bottom 50% | 1.21 | 0.17 | 2.61x10-01 | 0.87 | 1.68 |
| Persistent contact | PGS-SCZ top 5% vs. bottom 50% | 0.96 | 0.19 | 8.29x10-01 | 0.66 | 1.39 |
| Prolonged initial contact | PGS-BD top 5% vs. bottom 50% | 1.28 | 0.11 | 2.87x10-02 | 1.03 | 1.60 |
| Later re-entry | PGS-BD top 5% vs. bottom 50% | 1.16 | 0.17 | 3.71x10-01 | 0.84 | 1.62 |
| Persistent contact | PGS-BD top 5% vs. bottom 50% | 1.02 | 0.19 | 9.27x10-01 | 0.70 | 1.48 |
| Prolonged initial contact | PGS-AN top 5% vs. bottom 50% | 1.14 | 0.11 | 2.34x10-01 | 0.92 | 1.43 |
| Later re-entry | PGS-AN top 5% vs. bottom 50% | 1.07 | 0.17 | 6.84x10-01 | 0.77 | 1.50 |
| Persistent contact | PGS-AN top 5% vs. bottom 50% | 1.06 | 0.19 | 7.67x10-01 | 0.73 | 1.53 |
| Prolonged initial contact | PGS-ASD top 5% vs. bottom 50% | 1.17 | 0.11 | 1.56x10-01 | 0.94 | 1.47 |
| Later re-entry | PGS-ASD top 5% vs. bottom 50% | 1.24 | 0.16 | 1.85x10-01 | 0.90 | 1.71 |
| Persistent contact | PGS-ASD top 5% vs. bottom 50% | 0.98 | 0.19 | 9.11x10-01 | 0.68 | 1.41 |

*The brief contact class (class 1) was used as the reference category in multinomial regressions. Dark grey background represents results significant at the Bonferroni-adjusted alpha level of 0.00083.*

# STable 5. Mutually adjusted associations between PGS and trajectory class membership

| Trajectory class | PGS | OR | SE | p-value | Lower CI | Upper CI |
| --- | --- | --- | --- | --- | --- | --- |
| Prolonged initial contact | PGS-MDD | 0.95 | 0.03 | 8.58x10-02 | 0.91 | 1.01 |
| Later re-entry | PGS-MDD | 1.09 | 0.04 | 2.50x10-02 | 1.01 | 1.18 |
| Persistent contact | PGS-MDD | 0.96 | 0.04 | 3.37x10-01 | 0.88 | 1.04 |
| Prolonged initial contact | PGS-ADHD | 0.90 | 0.03 | 1.93x10-04 | 0.86 | 0.95 |
| Later re-entry | PGS-ADHD | 0.95 | 0.04 | 2.26x10-01 | 0.88 | 1.03 |
| Persistent contact | PGS-ADHD | 0.91 | 0.04 | 3.28x10-02 | 0.84 | 0.99 |
| Prolonged initial contact | PGS-ASD | 1.05 | 0.03 | 6.76x10-02 | 1.00 | 1.11 |
| Later re-entry | PGS-ASD | 1.08 | 0.04 | 5.38x10-02 | 1.00 | 1.17 |
| Persistent contact | PGS-ASD | 0.98 | 0.04 | 6.19x10-01 | 0.90 | 1.06 |
| Prolonged initial contact | PGS-BD | 1.05 | 0.03 | 5.71x10-02 | 1.00 | 1.11 |
| Later re-entry | PGS-BD | 0.95 | 0.04 | 2.56x10-01 | 0.88 | 1.03 |
| Persistent contact | PGS-BD | 0.99 | 0.04 | 8.96x10-01 | 0.91 | 1.09 |
| Prolonged initial contact | PGS-SCZ | 0.97 | 0.03 | 3.07x10-01 | 0.92 | 1.03 |
| Later re-entry | PGS-SCZ | 1.04 | 0.04 | 3.69x10-01 | 0.96 | 1.13 |
| Persistent contact | PGS-SCZ | 1.00 | 0.05 | 9.46x10-01 | 0.91 | 1.09 |
| Prolonged initial contact | PGS-AN | 1.06 | 0.03 | 2.81x10-02 | 1.01 | 1.11 |
| Later re-entry | PGS-AN | 1.00 | 0.04 | 9.94x10-01 | 0.93 | 1.08 |
| Persistent contact | PGS-AN | 1.13 | 0.04 | 2.64x10-03 | 1.04 | 1.22 |

*Results obtained from a single multinomial logistic regression model including all 6 PGS variables as well as an indicator variable for genotyping array (iPSYCH2012 vs. iPSYCH205), age at first depression contact, calendar year at first depression contact, and the first 5 principal components. The brief contact class (class 1) was used as the reference category. Dark grey background represents results significant at the Bonferroni-adjusted alpha level of 0.00083.*

# Stable 6. Frequency of treatment for other psychiatric diagnoses and continued treatment in primary care in the remaining follow-up in individuals in the brief contact class.

| **Outcome during follow-up** | **N = 6,876^1^** |
| --- | --- |
| Treatment for any other psychiatric disorder | 3,418 (49.7%) |
| F0: Organic disorder | 51 (0.7%) |
| F1: Mental and behavioural disorder due to psychoative substance use | 552 (8.0%) |
| F2: Schizophrenia, schizotypal and delusional disorder | 713 (10.4%) |
| F30-31: Mania/bipolar disorder | 211 (3.1%) |
| F4: Neurotic, stress-related and somatoform disorders | 1,830 (26.6%) |
| F50: Eating disorders | 318 (4.6%) |
| F51-F59: Behavioural syndromes associated with physiological disturbances and physical factors | 28 (0.4%) |
| F6: Personality disorder | 1,302 (18.9%) |
| F7: Mental retardation | 89 (1.3%) |
| F8: Disorders of psychological development | 252 (3.7%) |
| F90-98: Behavioural and emotional disorders with onset usually occurring in childhood and adolescence | 557 (8.1%) |
| F99: Unspecified mental disorder | 328 (4.8%) |
| Redeemed a prescription of antidepressants (any time after final discharge) | 5,072 (73.8%) |
| Redeemed a prescription of antidepressants (+6 months after final discharge) | 4,426 (64.4%) |
| Redeemed a prescription of antidepressants (+12 months after final discharge) | 4,049 (58.9%) |
| ^1^n (%) | |

***Treatment for other psychiatric disorders:*** *Information regarding treatment for other psychiatric disorders was taken from the Danish Psychiatric Central Research Register which includes diagnoses received within a Danish psychiatric hospital. Each diagnostic category was defined based on the codes from F disorders chapter (mental and behavioural disorders) in the 10^th^ edition of the International Classification of Diseases given at the discharge date of the hospital contact. We included all hospital contacts from the date that the individual was discharged for MDD for the last time up until the end of the 7-year follow-up period. Diagnostic categories are not mutually exclusive.*

***Continued treatment in primary care:*** *Continued treatment in primary care was indexed by redeeming a prescription of antidepressants in the remaining follow-up period after the final discharge from a psychiatric hospital for MDD. Three analyses were conducted: 1) including prescriptions redeemed any time after final discharge, 2) including prescriptions redeemed only 6 months or more after final discharge, and 3) including prescriptions redeemed only 12 months or more after final discharge.*

# STable 7. Associations between continuous polygenic scores (PGSs) and continued treatment in primary care in the remaining follow-up in individuals in the brief contact class.

***Any time after final discharge***

| Antidepressants | PGS | OR | SE | p-value | Lower CI | Upper CI |
| --- | --- | --- | --- | --- | --- | --- |
| Any time | PGS-MDD | 1.11 | 0.03 | 3.04x10-04 | 1.05 | 1.17 |
| Any time | PGS-ADHD | 1.01 | 0.03 | 8.55x10-01 | 0.95 | 1.06 |
| Any time | PGS-ASD | 1.03 | 0.03 | 2.80x10-01 | 0.98 | 1.09 |
| Any time | PGS-BD | 1.02 | 0.03 | 5.37x10-01 | 0.96 | 1.08 |
| Any time | PGS-SCZ | 1.01 | 0.03 | 8.50x10-01 | 0.95 | 1.06 |
| Any time | PGS-AN | 1.06 | 0.03 | 4.31x10-02 | 1.00 | 1.12 |

***+6 months after final discharge***

| Antidepressants | PGS | OR | SE | p-value | Lower CI | Upper CI |
| --- | --- | --- | --- | --- | --- | --- |
| +6 months | PGS-MDD | 1.10 | 0.03 | 1.05x10-04 | 1.05 | 1.16 |
| +6 months | PGS-ADHD | 1.00 | 0.03 | 8.71x10-01 | 0.95 | 1.05 |
| +6 months | PGS-ASD | 1.02 | 0.03 | 5.46x10-01 | 0.97 | 1.07 |
| +6 months | PGS-BD | 1.01 | 0.03 | 6.08x10-01 | 0.96 | 1.07 |
| +6 months | PGS-SCZ | 1.00 | 0.03 | 8.88x10-01 | 0.95 | 1.06 |
| +6 months | PGS-AN | 1.06 | 0.03 | 3.50x10-02 | 1.00 | 1.11 |

***+1 year after final discharge***

| Antidepressants | PGS | OR | SE | p-value | Lower CI | Upper CI |
| --- | --- | --- | --- | --- | --- | --- |
| +1 year | PGS-MDD | 1.10 | 0.03 | 1.64x10-04 | 1.05 | 1.15 |
| +1 year | PGS-ADHD | 0.99 | 0.02 | 7.47x10-01 | 0.94 | 1.04 |
| +1 year | PGS-ASD | 1.01 | 0.02 | 7.05x10-01 | 0.96 | 1.06 |
| +1 year | PGS-BD | 1.01 | 0.02 | 7.10x10-01 | 0.96 | 1.06 |
| +1 year | PGS-SCZ | 1.01 | 0.03 | 7.27x10-01 | 0.96 | 1.06 |
| +1 year | PGS-AN | 1.07 | 0.02 | 1.04x10-02 | 1.02 | 1.12 |

*Dark grey background represents results significant at the Bonferroni-adjusted alpha level of 0.00083.*

# STable 8. Associations between continuous polygenic scores (PGSs) and continued treatment in primary care in the remaining follow-up in individuals in the brief contact class – mutually adjusted models.

***Any time after final discharge***

| Antidepressants | PGS | OR | SE | p-value | Lower CI | Upper CI |
| --- | --- | --- | --- | --- | --- | --- |
| Any time | PGS-MDD | 1.11 | 0.03 | 7.18x10-04 | 1.04 | 1.18 |
| Any time | PGS-ADHD | 0.98 | 0.03 | 4.27x10-01 | 0.92 | 1.04 |
| Any time | PGS-ASD | 1.02 | 0.03 | 5.69x10-01 | 0.96 | 1.08 |
| Any time | PGS-BD | 1.00 | 0.03 | 9.64x10-01 | 0.94 | 1.06 |
| Any time | PGS-SCZ | 0.98 | 0.03 | 4.65x10-01 | 0.92 | 1.04 |
| Any time | PGS-AN | 1.04 | 0.03 | 1.40x10-01 | 0.99 | 1.10 |

***+6 months after final discharge***

| Antidepressants | PGS | OR | SE | p-value | Lower CI | Upper CI |
| --- | --- | --- | --- | --- | --- | --- |
| +6 months | PGS-MDD | 1.11 | 0.03 | 1.46x10-04 | 1.05 | 1.17 |
| +6 months | PGS-ADHD | 0.97 | 0.03 | 2.82x10-01 | 0.92 | 1.02 |
| +6 months | PGS-ASD | 1.00 | 0.03 | 8.87x10-01 | 0.95 | 1.06 |
| +6 months | PGS-BD | 1.00 | 0.03 | 8.73x10-01 | 0.94 | 1.05 |
| +6 months | PGS-SCZ | 0.98 | 0.03 | 4.66x10-01 | 0.92 | 1.04 |
| +6 months | PGS-AN | 1.04 | 0.03 | 1.21x10-01 | 0.99 | 1.10 |

***+1 year after final discharge***

| Antidepressants | PGS | OR | SE | p-value | Lower CI | Upper CI |
| --- | --- | --- | --- | --- | --- | --- |
| +1 year | PGS-MDD | 1.10 | 0.03 | 2.41x10-04 | 1.05 | 1.16 |
| +1 year | PGS-ADHD | 0.97 | 0.03 | 2.52x10-01 | 0.92 | 1.02 |
| +1 year | PGS-ASD | 1.00 | 0.03 | 9.35x10-01 | 0.95 | 1.05 |
| +1 year | PGS-BD | 0.99 | 0.03 | 6.88x10-01 | 0.94 | 1.04 |
| +1 year | PGS-SCZ | 0.99 | 0.03 | 6.65x10-01 | 0.93 | 1.04 |
| +1 year | PGS-AN | 1.05 | 0.03 | 4.27x10-02 | 1.00 | 1.11 |

*Dark grey background represents results significant at the Bonferroni-adjusted alpha level of 0.00083.*

# Stable 9. Associations between dichotomized (10%) polygenic scores (PGSs) and continued treatment in primary care in the remaining follow-up in individuals in the brief contact class.

***Any time after final discharge***

| Antidepressants | PGS | OR | SE | p-value | Lower CI | Upper CI |
| --- | --- | --- | --- | --- | --- | --- |
| Any time | PGS-MDD top 10% vs. bottom 10% | 1.31 | 0.13 | 3.40x10-02 | 1.02 | 1.68 |
| Any time | PGS-ADHD top 10% vs. bottom 10% | 1.04 | 0.12 | 7.45x10-01 | 0.82 | 1.33 |
| Any time | PGS-SCZ top 10% vs. bottom 10% | 1.05 | 0.13 | 7.27x10-01 | 0.81 | 1.35 |
| Any time | PGS-BD top 10% vs. bottom 10% | 1.15 | 0.13 | 2.86x10-01 | 0.89 | 1.48 |
| Any time | PGS-AN top 10% vs. bottom 10% | 1.36 | 0.13 | 1.55x10-02 | 1.06 | 1.75 |
| Any time | PGS-ASD top 10% vs. bottom 10% | 1.14 | 0.13 | 3.02x10-01 | 0.89 | 1.48 |
| Any time | PGS-MDD top 10% vs. bottom 90% | 1.16 | 0.10 | 1.31x10-01 | 0.96 | 1.40 |
| Any time | PGS-ADHD top 10% vs. bottom 90% | 1.00 | 0.09 | 9.80x10-01 | 0.83 | 1.20 |
| Any time | PGS-SCZ top 10% vs. bottom 90% | 1.15 | 0.10 | 1.37x10-01 | 0.96 | 1.39 |
| Any time | PGS-BD top 10% vs. bottom 90% | 1.16 | 0.10 | 1.12x10-01 | 0.97 | 1.41 |
| Any time | PGS-AN top 10% vs. bottom 90% | 1.18 | 0.10 | 8.32x10-02 | 0.98 | 1.43 |
| Any time | PGS-ASD top 10% vs. bottom 90% | 1.24 | 0.10 | 2.56x10-02 | 1.03 | 1.51 |
| Any time | PGS-MDD top 10% vs. bottom 50% | 1.25 | 0.10 | 2.50x10-02 | 1.03 | 1.52 |
| Any time | PGS-ADHD top 10% vs. bottom 50% | 1.01 | 0.10 | 9.16x10-01 | 0.84 | 1.22 |
| Any time | PGS-SCZ top 10% vs. bottom 50% | 1.16 | 0.10 | 1.36x10-01 | 0.96 | 1.42 |
| Any time | PGS-BD top 10% vs. bottom 50% | 1.14 | 0.10 | 1.77x10-01 | 0.94 | 1.39 |
| Any time | PGS-AN top 10% vs. bottom 50% | 1.19 | 0.10 | 8.43x10-02 | 0.98 | 1.45 |
| Any time | PGS-ASD top 10% vs. bottom 50% | 1.23 | 0.10 | 3.96x10-02 | 1.01 | 1.50 |

***+6 months after final discharge***

| Antidepressants | PGS | OR | SE | p-value | Lower CI | Upper CI |
| --- | --- | --- | --- | --- | --- | --- |
| +6 months | PGS-MDD top 10% vs. bottom 10% | 1.34 | 0.12 | 1.06x10-02 | 1.07 | 1.69 |
| +6 months | PGS-ADHD top 10% vs. bottom 10% | 1.01 | 0.11 | 9.32x10-01 | 0.81 | 1.26 |
| +6 months | PGS-SCZ top 10% vs. bottom 10% | 1.05 | 0.12 | 6.85x10-01 | 0.83 | 1.32 |
| +6 months | PGS-BD top 10% vs. bottom 10% | 1.11 | 0.12 | 3.67x10-01 | 0.88 | 1.39 |
| +6 months | PGS-AN top 10% vs. bottom 10% | 1.28 | 0.12 | 3.52x10-02 | 1.02 | 1.60 |
| +6 months | PGS-ASD top 10% vs. bottom 10% | 1.08 | 0.12 | 4.93x10-01 | 0.86 | 1.37 |
| +6 months | PGS-MDD top 10% vs. bottom 90% | 1.14 | 0.09 | 1.37x10-01 | 0.96 | 1.35 |
| +6 months | PGS-ADHD top 10% vs. bottom 90% | 1.01 | 0.09 | 9.14x10-01 | 0.85 | 1.19 |
| +6 months | PGS-SCZ top 10% vs. bottom 90% | 1.20 | 0.09 | 3.59x10-02 | 1.01 | 1.43 |
| +6 months | PGS-BD top 10% vs. bottom 90% | 1.09 | 0.09 | 3.31x10-01 | 0.92 | 1.29 |
| +6 months | PGS-AN top 10% vs. bottom 90% | 1.15 | 0.09 | 9.79x10-02 | 0.98 | 1.37 |
| +6 months | PGS-ASD top 10% vs. bottom 90% | 1.20 | 0.09 | 3.44x10-02 | 1.01 | 1.43 |
| +6 months | PGS-MDD top 10% vs. bottom 50% | 1.23 | 0.09 | 2.19x10-02 | 1.03 | 1.47 |
| +6 months | PGS-ADHD top 10% vs. bottom 50% | 1.02 | 0.09 | 8.67x10-01 | 0.85 | 1.21 |
| +6 months | PGS-SCZ top 10% vs. bottom 50% | 1.18 | 0.09 | 6.87x10-02 | 0.99 | 1.42 |
| +6 months | PGS-BD top 10% vs. bottom 50% | 1.09 | 0.09 | 3.56x10-01 | 0.91 | 1.30 |
| +6 months | PGS-AN top 10% vs. bottom 50% | 1.18 | 0.09 | 6.91x10-02 | 0.99 | 1.41 |
| +6 months | PGS-ASD top 10% vs. bottom 50% | 1.18 | 0.09 | 7.38x10-02 | 0.99 | 1.41 |

***+1 year after final discharge***

| Antidepressants | PGS | OR | SE | p-value | Lower CI | Upper CI |
| --- | --- | --- | --- | --- | --- | --- |
| +1 year | PGS-MDD top 10% vs. bottom 10% | 1.36 | 0.11 | 6.11x10-03 | 1.09 | 1.70 |
| +1 year | PGS-ADHD top 10% vs. bottom 10% | 0.99 | 0.11 | 9.55x10-01 | 0.80 | 1.24 |
| +1 year | PGS-SCZ top 10% vs. bottom 10% | 1.10 | 0.11 | 4.16x10-01 | 0.88 | 1.37 |
| +1 year | PGS-BD top 10% vs. bottom 10% | 1.13 | 0.11 | 2.69x10-01 | 0.91 | 1.41 |
| +1 year | PGS-AN top 10% vs. bottom 10% | 1.34 | 0.11 | 9.69x10-03 | 1.07 | 1.67 |
| +1 year | PGS-ASD top 10% vs. bottom 10% | 1.05 | 0.11 | 6.80x10-01 | 0.84 | 1.31 |
| +1 year | PGS-MDD top 10% vs. bottom 90% | 1.20 | 0.08 | 2.97x10-02 | 1.02 | 1.42 |
| +1 year | PGS-ADHD top 10% vs. bottom 90% | 0.99 | 0.08 | 9.29x10-01 | 0.84 | 1.17 |
| +1 year | PGS-SCZ top 10% vs. bottom 90% | 1.16 | 0.08 | 7.89x10-02 | 0.98 | 1.37 |
| +1 year | PGS-BD top 10% vs. bottom 90% | 1.09 | 0.08 | 3.02x10-01 | 0.93 | 1.28 |
| +1 year | PGS-AN top 10% vs. bottom 90% | 1.20 | 0.08 | 3.01x10-02 | 1.02 | 1.42 |
| +1 year | PGS-ASD top 10% vs. bottom 90% | 1.14 | 0.08 | 1.07x10-01 | 0.97 | 1.35 |
| +1 year | PGS-MDD top 10% vs. bottom 50% | 1.27 | 0.09 | 7.07x10-03 | 1.07 | 1.50 |
| +1 year | PGS-ADHD top 10% vs. bottom 50% | 1.00 | 0.09 | 9.96x10-01 | 0.85 | 1.19 |
| +1 year | PGS-SCZ top 10% vs. bottom 50% | 1.14 | 0.09 | 1.40x10-01 | 0.96 | 1.35 |
| +1 year | PGS-BD top 10% vs. bottom 50% | 1.09 | 0.09 | 3.40x10-01 | 0.92 | 1.29 |
| +1 year | PGS-AN top 10% vs. bottom 50% | 1.23 | 0.09 | 1.59x10-02 | 1.04 | 1.47 |
| +1 year | PGS-ASD top 10% vs. bottom 50% | 1.13 | 0.09 | 1.69x10-01 | 0.95 | 1.34 |

*Dark grey background represents results significant at the Bonferroni-adjusted alpha level of 0.00083.*

# STable 10. Associations between dichotomized (5%) polygenic scores (PGSs) and continued treatment in primary care in the remaining follow-up in individuals in the brief contact class.

***Any time after final discharge***

| Antidepressants | PGS | OR | SE | p-value | Lower CI | Upper CI |
| --- | --- | --- | --- | --- | --- | --- |
| Any time | PGS-MDD top 5% vs. bottom 5% | 1.16 | 0.18 | 4.25x10-01 | 0.81 | 1.65 |
| Any time | PGS-ADHD top 5% vs. bottom 5% | 1.27 | 0.18 | 1.72x10-01 | 0.90 | 1.81 |
| Any time | PGS-SCZ top 5% vs. bottom 5% | 1.03 | 0.18 | 8.88x10-01 | 0.72 | 1.46 |
| Any time | PGS-BD top 5% vs. bottom 5% | 1.17 | 0.19 | 4.10x10-01 | 0.81 | 1.69 |
| Any time | PGS-AN top 5% vs. bottom 5% | 1.19 | 0.18 | 3.31x10-01 | 0.84 | 1.71 |
| Any time | PGS-ASD top 5% vs. bottom 5% | 1.20 | 0.19 | 3.32x10-01 | 0.83 | 1.73 |
| Any time | PGS-MDD top 5% vs. bottom 95% | 1.09 | 0.13 | 5.24x10-01 | 0.84 | 1.41 |
| Any time | PGS-ADHD top 5% vs. bottom 95% | 1.16 | 0.13 | 2.68x10-01 | 0.90 | 1.50 |
| Any time | PGS-SCZ top 5% vs. bottom 95% | 0.99 | 0.13 | 9.24x10-01 | 0.77 | 1.27 |
| Any time | PGS-BD top 5% vs. bottom 95% | 1.25 | 0.13 | 9.92x10-02 | 0.96 | 1.63 |
| Any time | PGS-AN top 5% vs. bottom 95% | 1.13 | 0.13 | 3.46x10-01 | 0.88 | 1.47 |
| Any time | PGS-ASD top 5% vs. bottom 95% | 1.24 | 0.13 | 1.11x10-01 | 0.96 | 1.62 |
| Any time | PGS-MDD top 5% vs. bottom 50% | 1.19 | 0.13 | 1.97x10-01 | 0.92 | 1.55 |
| Any time | PGS-ADHD top 5% vs. bottom 50% | 1.16 | 0.13 | 2.64x10-01 | 0.90 | 1.52 |
| Any time | PGS-SCZ top 5% vs. bottom 50% | 1.01 | 0.13 | 9.53x10-01 | 0.78 | 1.31 |
| Any time | PGS-BD top 5% vs. bottom 50% | 1.24 | 0.14 | 1.20x10-01 | 0.95 | 1.63 |
| Any time | PGS-AN top 5% vs. bottom 50% | 1.16 | 0.14 | 2.81x10-01 | 0.89 | 1.51 |
| Any time | PGS-ASD top 5% vs. bottom 50% | 1.24 | 0.14 | 1.24x10-01 | 0.95 | 1.63 |

***+6 months after final discharge***

| Antidepressants | PGS | OR | SE | p-value | Lower CI | Upper CI |
| --- | --- | --- | --- | --- | --- | --- |
| +6 months | PGS-MDD top 5% vs. bottom 5% | 1.40 | 0.17 | 4.32x10-02 | 1.01 | 1.94 |
| +6 months | PGS-ADHD top 5% vs. bottom 5% | 1.25 | 0.16 | 1.69x10-01 | 0.91 | 1.73 |
| +6 months | PGS-SCZ top 5% vs. bottom 5% | 0.93 | 0.17 | 6.49x10-01 | 0.67 | 1.28 |
| +6 months | PGS-BD top 5% vs. bottom 5% | 1.15 | 0.17 | 3.96x10-01 | 0.83 | 1.61 |
| +6 months | PGS-AN top 5% vs. bottom 5% | 1.23 | 0.17 | 2.03x10-01 | 0.89 | 1.71 |
| +6 months | PGS-ASD top 5% vs. bottom 5% | 1.09 | 0.17 | 6.03x10-01 | 0.79 | 1.51 |
| +6 months | PGS-MDD top 5% vs. bottom 95% | 1.19 | 0.12 | 1.52x10-01 | 0.94 | 1.51 |
| +6 months | PGS-ADHD top 5% vs. bottom 95% | 1.16 | 0.12 | 2.17x10-01 | 0.92 | 1.47 |
| +6 months | PGS-SCZ top 5% vs. bottom 95% | 0.98 | 0.12 | 8.90x10-01 | 0.78 | 1.24 |
| +6 months | PGS-BD top 5% vs. bottom 95% | 1.17 | 0.12 | 1.84x10-01 | 0.93 | 1.49 |
| +6 months | PGS-AN top 5% vs. bottom 95% | 1.12 | 0.12 | 3.55x10-01 | 0.89 | 1.41 |
| +6 months | PGS-ASD top 5% vs. bottom 95% | 1.12 | 0.12 | 3.52x10-01 | 0.89 | 1.42 |
| +6 months | PGS-MDD top 5% vs. bottom 50% | 1.29 | 0.12 | 3.80x10-02 | 1.02 | 1.65 |
| +6 months | PGS-ADHD top 5% vs. bottom 50% | 1.16 | 0.12 | 2.25x10-01 | 0.92 | 1.48 |
| +6 months | PGS-SCZ top 5% vs. bottom 50% | 0.98 | 0.12 | 8.62x10-01 | 0.77 | 1.24 |
| +6 months | PGS-BD top 5% vs. bottom 50% | 1.17 | 0.12 | 1.90x10-01 | 0.93 | 1.50 |
| +6 months | PGS-AN top 5% vs. bottom 50% | 1.15 | 0.12 | 2.38x10-01 | 0.91 | 1.47 |
| +6 months | PGS-ASD top 5% vs. bottom 50% | 1.10 | 0.12 | 4.25x10-01 | 0.87 | 1.40 |

***+1 year after final discharge***

| Antidepressants | PGS | OR | SE | p-value | Lower CI | Upper CI |
| --- | --- | --- | --- | --- | --- | --- |
| +1 year | PGS-MDD top 5% vs. bottom 5% | 1.41 | 0.16 | 3.61x10-02 | 1.02 | 1.93 |
| +1 year | PGS-ADHD top 5% vs. bottom 5% | 1.27 | 0.16 | 1.37x10-01 | 0.93 | 1.73 |
| +1 year | PGS-SCZ top 5% vs. bottom 5% | 1.10 | 0.16 | 5.72x10-01 | 0.80 | 1.50 |
| +1 year | PGS-BD top 5% vs. bottom 5% | 1.13 | 0.16 | 4.47x10-01 | 0.82 | 1.56 |
| +1 year | PGS-AN top 5% vs. bottom 5% | 1.39 | 0.16 | 4.23x10-02 | 1.01 | 1.90 |
| +1 year | PGS-ASD top 5% vs. bottom 5% | 0.96 | 0.16 | 8.21x10-01 | 0.70 | 1.32 |
| +1 year | PGS-MDD top 5% vs. bottom 95% | 1.24 | 0.12 | 6.67x10-02 | 0.99 | 1.56 |
| +1 year | PGS-ADHD top 5% vs. bottom 95% | 1.10 | 0.11 | 4.05x10-01 | 0.88 | 1.38 |
| +1 year | PGS-SCZ top 5% vs. bottom 95% | 1.08 | 0.12 | 5.28x10-01 | 0.86 | 1.35 |
| +1 year | PGS-BD top 5% vs. bottom 95% | 1.15 | 0.12 | 2.36x10-01 | 0.92 | 1.44 |
| +1 year | PGS-AN top 5% vs. bottom 95% | 1.27 | 0.12 | 4.29x10-02 | 1.01 | 1.60 |
| +1 year | PGS-ASD top 5% vs. bottom 95% | 1.02 | 0.11 | 8.48x10-01 | 0.82 | 1.28 |
| +1 year | PGS-MDD top 5% vs. bottom 50% | 1.32 | 0.12 | 2.05x10-02 | 1.05 | 1.67 |
| +1 year | PGS-ADHD top 5% vs. bottom 50% | 1.11 | 0.12 | 3.88x10-01 | 0.88 | 1.40 |
| +1 year | PGS-SCZ top 5% vs. bottom 50% | 1.06 | 0.12 | 6.16x10-01 | 0.84 | 1.34 |
| +1 year | PGS-BD top 5% vs. bottom 50% | 1.15 | 0.12 | 2.45x10-01 | 0.91 | 1.45 |
| +1 year | PGS-AN top 5% vs. bottom 50% | 1.32 | 0.12 | 2.10x10-02 | 1.04 | 1.67 |
| +1 year | PGS-ASD top 5% vs. bottom 50% | 1.01 | 0.12 | 9.02x10-01 | 0.81 | 1.28 |

*Dark grey background represents results significant at the Bonferroni-adjusted alpha level of 0.00083.*

# STable 11. Association between continuous polygenic scores (PGSs) and treatment for other psychiatric disorders in secondary care in the remaining follow-up in individuals in the brief contact class.

***ICD-10 F1 disorders - Mental and behavioural disorder due to psychoative substance use***

| ICD-10 disorders | PGS | OR | SE | p-value | Lower CI | Upper CI |
| --- | --- | --- | --- | --- | --- | --- |
| F1 | PGS-MDD | 1.17 | 0.05 | 5.75x10-04 | 1.07 | 1.28 |
| F1 | PGS-ADHD | 1.30 | 0.05 | 9.51x10-09 | 1.19 | 1.42 |
| F1 | PGS-ASD | 1.01 | 0.05 | 8.94x10-01 | 0.92 | 1.10 |
| F1 | PGS-BD | 1.11 | 0.05 | 1.86x10-02 | 1.02 | 1.21 |
| F1 | PGS-SCZ | 1.18 | 0.05 | 3.28x10-04 | 1.08 | 1.29 |
| F1 | PGS-AN | 0.99 | 0.05 | 8.21x10-01 | 0.91 | 1.08 |

***ICD-10 F2 disorders - Schizophrenia. schizotypal and delusional disorder***

| ICD-10 disorders | PGS | OR | SE | p-value | Lower CI | Upper CI |
| --- | --- | --- | --- | --- | --- | --- |
| F2 | PGS-MDD | 1.09 | 0.05 | 5.02x10-02 | 1.00 | 1.19 |
| F2 | PGS-ADHD | 1.06 | 0.05 | 2.22x10-01 | 0.97 | 1.15 |
| F2 | PGS-ASD | 1.15 | 0.05 | 2.15x10-03 | 1.05 | 1.26 |
| F2 | PGS-BD | 1.06 | 0.05 | 1.74x10-01 | 0.97 | 1.16 |
| F2 | PGS-SCZ | 1.27 | 0.05 | 2.26x10-07 | 1.16 | 1.38 |
| F2 | PGS-AN | 1.11 | 0.05 | 2.03x10-02 | 1.02 | 1.21 |

***ICD-10 F4 disorders - Neurotic, stress-related and somatoform disorders***

| ICD-10 disorders | PGS | OR | SE | p-value | Lower CI | Upper CI |
| --- | --- | --- | --- | --- | --- | --- |
| F4 | PGS-MDD | 1.13 | 0.03 | 9.14x10-06 | 1.07 | 1.19 |
| F4 | PGS-ADHD | 1.08 | 0.03 | 3.03x10-03 | 1.03 | 1.14 |
| F4 | PGS-ASD | 0.99 | 0.03 | 7.37x10-01 | 0.94 | 1.05 |
| F4 | PGS-BD | 1.04 | 0.03 | 1.70x10-01 | 0.98 | 1.10 |
| F4 | PGS-SCZ | 1.03 | 0.03 | 2.95x10-01 | 0.98 | 1.09 |
| F4 | PGS-AN | 1.06 | 0.03 | 2.94x10-02 | 1.01 | 1.12 |

***ICD-10 F6 disorders - Personality disorder***

| ICD-10 disorders | PGS | OR | SE | p-value | Lower CI | Upper CI |
| --- | --- | --- | --- | --- | --- | --- |
| F6 | PGS-MDD | 1.15 | 0.03 | 1.14x10-05 | 1.08 | 1.22 |
| F6 | PGS-ADHD | 1.11 | 0.03 | 8.41x10-04 | 1.04 | 1.18 |
| F6 | PGS-ASD | 1.02 | 0.03 | 4.52x10-01 | 0.96 | 1.09 |
| F6 | PGS-BD | 1.02 | 0.03 | 5.22x10-01 | 0.96 | 1.08 |
| F6 | PGS-SCZ | 1.06 | 0.03 | 7.85x10-02 | 0.99 | 1.12 |
| F6 | PGS-AN | 1.04 | 0.03 | 2.57x10-01 | 0.97 | 1.10 |

***ICD-10 F90-F98 disorders - Behavioural and emotional disorders with onset usually occurring in childhood and adolescence***

| ICD-10 disorders | PGS | OR | SE | p-value | Lower CI | Upper CI |
| --- | --- | --- | --- | --- | --- | --- |
| F90-F98 | PGS-MDD | 1.11 | 0.05 | 2.25x10-02 | 1.01 | 1.21 |
| F90-F98 | PGS-ADHD | 1.40 | 0.05 | 1.59x10-13 | 1.28 | 1.53 |
| F90-F98 | PGS-ASD | 1.09 | 0.05 | 5.76x10-02 | 1.00 | 1.19 |
| F90-F98 | PGS-BD | 0.99 | 0.05 | 8.49x10-01 | 0.91 | 1.08 |
| F90-F98 | PGS-SCZ | 1.03 | 0.05 | 5.66x10-01 | 0.94 | 1.12 |
| F90-F98 | PGS-AN | 0.99 | 0.04 | 9.09x10-01 | 0.91 | 1.09 |

# STable 12. Association between dichotomized (10%) polygenic scores (PGSs) and treatment for other psychiatric disorders in secondary care in the remaining follow-up in individuals in the brief contact class.

***ICD-10 F1 disorders - Mental and behavioural disorder due to psychoative substance use***

| ICD-10 disorders | PGS | OR | SE | p-value | Lower CI | Upper CI |
| --- | --- | --- | --- | --- | --- | --- |
| F1 | PGS-MDD top 10% vs. bottom 10% | 1.77 | 0.21 | 7.71x10-03 | 1.17 | 2.70 |
| F1 | PGS-ADHD top 10% vs. bottom 10% | 2.52 | 0.21 | 8.03x10-06 | 1.69 | 3.82 |
| F1 | PGS-SCZ top 10% vs. bottom 10% | 1.94 | 0.21 | 1.85x10-03 | 1.29 | 2.97 |
| F1 | PGS-BD top 10% vs. bottom 10% | 1.16 | 0.20 | 4.56x10-01 | 0.78 | 1.73 |
| F1 | PGS-AN top 10% vs. bottom 10% | 1.10 | 0.21 | 6.31x10-01 | 0.74 | 1.66 |
| F1 | PGS-ASD top 10% vs. bottom 10% | 1.05 | 0.20 | 8.01x10-01 | 0.71 | 1.57 |
| F1 | PGS-MDD top 10% vs. bottom 90% | 1.21 | 0.14 | 1.68x10-01 | 0.91 | 1.59 |
| F1 | PGS-ADHD top 10% vs. bottom 90% | 1.82 | 0.13 | 2.66x10-06 | 1.41 | 2.33 |
| F1 | PGS-SCZ top 10% vs. bottom 90% | 1.29 | 0.14 | 6.24x10-02 | 0.98 | 1.68 |
| F1 | PGS-BD top 10% vs. bottom 90% | 1.10 | 0.14 | 5.27x10-01 | 0.82 | 1.44 |
| F1 | PGS-AN top 10% vs. bottom 90% | 1.05 | 0.15 | 7.58x10-01 | 0.78 | 1.38 |
| F1 | PGS-ASD top 10% vs. bottom 90% | 1.06 | 0.15 | 7.09x10-01 | 0.78 | 1.40 |
| F1 | PGS-MDD top 10% vs. bottom 50% | 1.38 | 0.15 | 2.89x10-02 | 1.03 | 1.84 |
| F1 | PGS-ADHD top 10% vs. bottom 50% | 2.12 | 0.14 | 4.89x10-08 | 1.61 | 2.77 |
| F1 | PGS-SCZ top 10% vs. bottom 50% | 1.55 | 0.15 | 2.61x10-03 | 1.16 | 2.06 |
| F1 | PGS-BD top 10% vs. bottom 50% | 1.13 | 0.15 | 4.26x10-01 | 0.83 | 1.51 |
| F1 | PGS-AN top 10% vs. bottom 50% | 0.99 | 0.15 | 9.39x10-01 | 0.73 | 1.32 |
| F1 | PGS-ASD top 10% vs. bottom 50% | 0.98 | 0.15 | 9.03x10-01 | 0.72 | 1.32 |

***ICD-10 F2 disorders - Schizophrenia. schizotypal and delusional disorder***

| ICD-10 disorders | PGS | OR | SE | p-value | Lower CI | Upper CI |
| --- | --- | --- | --- | --- | --- | --- |
| F2 | PGS-MDD top 10% vs. bottom 10% | 1.44 | 0.21 | 7.91x10-02 | 0.96 | 2.18 |
| F2 | PGS-ADHD top 10% vs. bottom 10% | 1.01 | 0.19 | 9.48x10-01 | 0.70 | 1.47 |
| F2 | PGS-SCZ top 10% vs. bottom 10% | 2.70 | 0.22 | 7.91x10-06 | 1.76 | 4.22 |
| F2 | PGS-BD top 10% vs. bottom 10% | 1.23 | 0.21 | 3.12x10-01 | 0.82 | 1.85 |
| F2 | PGS-AN top 10% vs. bottom 10% | 1.62 | 0.20 | 1.35x10-02 | 1.11 | 2.40 |
| F2 | PGS-ASD top 10% vs. bottom 10% | 1.25 | 0.20 | 2.64x10-01 | 0.85 | 1.83 |
| F2 | PGS-MDD top 10% vs. bottom 90% | 1.20 | 0.14 | 1.97x10-01 | 0.90 | 1.58 |
| F2 | PGS-ADHD top 10% vs. bottom 90% | 1.17 | 0.14 | 2.72x10-01 | 0.88 | 1.54 |
| F2 | PGS-SCZ top 10% vs. bottom 90% | 1.56 | 0.13 | 8.20x10-04 | 1.19 | 2.01 |
| F2 | PGS-BD top 10% vs. bottom 90% | 0.99 | 0.15 | 9.36x10-01 | 0.73 | 1.31 |
| F2 | PGS-AN top 10% vs. bottom 90% | 1.65 | 0.13 | 1.39x10-04 | 1.27 | 2.12 |
| F2 | PGS-ASD top 10% vs. bottom 90% | 1.14 | 0.14 | 3.58x10-01 | 0.85 | 1.50 |
| F2 | PGS-MDD top 10% vs. bottom 50% | 1.18 | 0.15 | 2.70x10-01 | 0.87 | 1.57 |
| F2 | PGS-ADHD top 10% vs. bottom 50% | 1.21 | 0.15 | 2.12x10-01 | 0.89 | 1.62 |
| F2 | PGS-SCZ top 10% vs. bottom 50% | 1.82 | 0.14 | 3.17x10-05 | 1.37 | 2.40 |
| F2 | PGS-BD top 10% vs. bottom 50% | 1.10 | 0.16 | 5.46x10-01 | 0.80 | 1.48 |
| F2 | PGS-AN top 10% vs. bottom 50% | 1.58 | 0.14 | 7.53x10-04 | 1.20 | 2.05 |
| F2 | PGS-ASD top 10% vs. bottom 50% | 1.31 | 0.15 | 7.01x10-02 | 0.97 | 1.75 |

***ICD-10 F4 disorders - Neurotic, stress-related and somatoform disorders***

| ICD-10 disorders | PGS | OR | SE | p-value | Lower CI | Upper CI |
| --- | --- | --- | --- | --- | --- | --- |
| F4 | PGS-MDD top 10% vs. bottom 10% | 1.67 | 0.12 | 4.03x10-05 | 1.31 | 2.13 |
| F4 | PGS-ADHD top 10% vs. bottom 10% | 1.33 | 0.12 | 2.13x10-02 | 1.04 | 1.69 |
| F4 | PGS-SCZ top 10% vs. bottom 10% | 1.04 | 0.12 | 7.63x10-01 | 0.82 | 1.32 |
| F4 | PGS-BD top 10% vs. bottom 10% | 1.14 | 0.13 | 2.80x10-01 | 0.90 | 1.46 |
| F4 | PGS-AN top 10% vs. bottom 10% | 1.24 | 0.12 | 7.90x10-02 | 0.98 | 1.58 |
| F4 | PGS-ASD top 10% vs. bottom 10% | 1.00 | 0.12 | 9.77x10-01 | 0.78 | 1.27 |
| F4 | PGS-MDD top 10% vs. bottom 90% | 1.35 | 0.09 | 6.04x10-04 | 1.13 | 1.60 |
| F4 | PGS-ADHD top 10% vs. bottom 90% | 1.20 | 0.09 | 4.33x10-02 | 1.00 | 1.42 |
| F4 | PGS-SCZ top 10% vs. bottom 90% | 1.08 | 0.09 | 3.76x10-01 | 0.91 | 1.29 |
| F4 | PGS-BD top 10% vs. bottom 90% | 1.07 | 0.09 | 4.64x10-01 | 0.89 | 1.27 |
| F4 | PGS-AN top 10% vs. bottom 90% | 1.12 | 0.09 | 2.05x10-01 | 0.94 | 1.33 |
| F4 | PGS-ASD top 10% vs. bottom 90% | 1.03 | 0.09 | 7.40x10-01 | 0.86 | 1.23 |
| F4 | PGS-MDD top 10% vs. bottom 50% | 1.43 | 0.09 | 7.60x10-05 | 1.20 | 1.71 |
| F4 | PGS-ADHD top 10% vs. bottom 50% | 1.26 | 0.09 | 1.22x10-02 | 1.05 | 1.51 |
| F4 | PGS-SCZ top 10% vs. bottom 50% | 1.13 | 0.09 | 2.04x10-01 | 0.94 | 1.35 |
| F4 | PGS-BD top 10% vs. bottom 50% | 1.06 | 0.09 | 5.17x10-01 | 0.88 | 1.28 |
| F4 | PGS-AN top 10% vs. bottom 50% | 1.15 | 0.09 | 1.33x10-01 | 0.96 | 1.38 |
| F4 | PGS-ASD top 10% vs. bottom 50% | 1.03 | 0.09 | 7.65x10-01 | 0.85 | 1.24 |

***ICD-10 F6 disorders - Personality disorder***

| ICD-10 disorders | PGS | OR | SE | p-value | Lower CI | Upper CI |
| --- | --- | --- | --- | --- | --- | --- |
| F6 | PGS-MDD top 10% vs. bottom 10% | 1.23 | 0.14 | 1.46x10-01 | 0.93 | 1.62 |
| F6 | PGS-ADHD top 10% vs. bottom 10% | 1.41 | 0.14 | 1.43x10-02 | 1.07 | 1.86 |
| F6 | PGS-SCZ top 10% vs. bottom 10% | 1.17 | 0.14 | 2.55x10-01 | 0.89 | 1.54 |
| F6 | PGS-BD top 10% vs. bottom 10% | 1.08 | 0.14 | 5.78x10-01 | 0.82 | 1.43 |
| F6 | PGS-AN top 10% vs. bottom 10% | 1.15 | 0.14 | 3.32x10-01 | 0.87 | 1.51 |
| F6 | PGS-ASD top 10% vs. bottom 10% | 1.20 | 0.14 | 1.98x10-01 | 0.91 | 1.58 |
| F6 | PGS-MDD top 10% vs. bottom 90% | 1.05 | 0.10 | 6.17x10-01 | 0.86 | 1.28 |
| F6 | PGS-ADHD top 10% vs. bottom 90% | 1.24 | 0.10 | 2.86x10-02 | 1.02 | 1.50 |
| F6 | PGS-SCZ top 10% vs. bottom 90% | 1.16 | 0.10 | 1.41x10-01 | 0.95 | 1.41 |
| F6 | PGS-BD top 10% vs. bottom 90% | 1.09 | 0.10 | 4.14x10-01 | 0.89 | 1.33 |
| F6 | PGS-AN top 10% vs. bottom 90% | 1.11 | 0.10 | 2.90x10-01 | 0.91 | 1.35 |
| F6 | PGS-ASD top 10% vs. bottom 90% | 1.08 | 0.10 | 4.36x10-01 | 0.88 | 1.32 |
| F6 | PGS-MDD top 10% vs. bottom 50% | 1.23 | 0.11 | 5.04x10-02 | 1.00 | 1.52 |
| F6 | PGS-ADHD top 10% vs. bottom 50% | 1.32 | 0.10 | 7.57x10-03 | 1.07 | 1.61 |
| F6 | PGS-SCZ top 10% vs. bottom 50% | 1.24 | 0.11 | 4.36x10-02 | 1.00 | 1.52 |
| F6 | PGS-BD top 10% vs. bottom 50% | 1.10 | 0.11 | 3.98x10-01 | 0.88 | 1.35 |
| F6 | PGS-AN top 10% vs. bottom 50% | 1.12 | 0.11 | 2.89x10-01 | 0.91 | 1.37 |
| F6 | PGS-ASD top 10% vs. bottom 50% | 1.09 | 0.11 | 4.14x10-01 | 0.88 | 1.34 |

***ICD-10 F90-F98 disorders - Behavioural and emotional disorders with onset usually occurring in childhood and adolescence***

| ICD-10 disorders | PGS | OR | SE | p-value | Lower CI | Upper CI |
| --- | --- | --- | --- | --- | --- | --- |
| F90-F98 | PGS-MDD top 10% vs. bottom 10% | 1.26 | 0.19 | 2.43x10-01 | 0.86 | 1.84 |
| F90-F98 | PGS-ADHD top 10% vs. bottom 10% | 2.94 | 0.20 | 1.39x10-07 | 1.98 | 4.43 |
| F90-F98 | PGS-SCZ top 10% vs. bottom 10% | 1.09 | 0.20 | 6.77x10-01 | 0.74 | 1.61 |
| F90-F98 | PGS-BD top 10% vs. bottom 10% | 1.00 | 0.21 | 9.84x10-01 | 0.66 | 1.51 |
| F90-F98 | PGS-AN top 10% vs. bottom 10% | 1.18 | 0.20 | 4.12x10-01 | 0.80 | 1.75 |
| F90-F98 | PGS-ASD top 10% vs. bottom 10% | 1.66 | 0.21 | 1.35x10-02 | 1.12 | 2.51 |
| F90-F98 | PGS-MDD top 10% vs. bottom 90% | 1.34 | 0.14 | 3.06x10-02 | 1.02 | 1.74 |
| F90-F98 | PGS-ADHD top 10% vs. bottom 90% | 2.06 | 0.12 | 4.64x10-09 | 1.61 | 2.62 |
| F90-F98 | PGS-SCZ top 10% vs. bottom 90% | 1.09 | 0.14 | 5.72x10-01 | 0.81 | 1.43 |
| F90-F98 | PGS-BD top 10% vs. bottom 90% | 0.95 | 0.15 | 7.26x10-01 | 0.70 | 1.26 |
| F90-F98 | PGS-AN top 10% vs. bottom 90% | 1.16 | 0.14 | 2.97x10-01 | 0.87 | 1.53 |
| F90-F98 | PGS-ASD top 10% vs. bottom 90% | 1.28 | 0.14 | 6.96x10-02 | 0.97 | 1.66 |
| F90-F98 | PGS-MDD top 10% vs. bottom 50% | 1.42 | 0.14 | 1.50x10-02 | 1.06 | 1.88 |
| F90-F98 | PGS-ADHD top 10% vs. bottom 50% | 2.55 | 0.13 | 3.09x10-12 | 1.95 | 3.31 |
| F90-F98 | PGS-SCZ top 10% vs. bottom 50% | 1.13 | 0.15 | 4.19x10-01 | 0.83 | 1.51 |
| F90-F98 | PGS-BD top 10% vs. bottom 50% | 0.97 | 0.16 | 8.52x10-01 | 0.71 | 1.31 |
| F90-F98 | PGS-AN top 10% vs. bottom 50% | 1.13 | 0.15 | 4.07x10-01 | 0.84 | 1.51 |
| F90-F98 | PGS-ASD top 10% vs. bottom 50% | 1.32 | 0.14 | 5.14x10-02 | 0.99 | 1.74 |

# STable 13. Association between dichotomized (5%) polygenic scores (PGSs) and treatment for other psychiatric disorders in secondary care in the remaining follow-up in individuals in the brief contact class.

***ICD-10 F1 disorders - Mental and behavioural disorder due to psychoative substance use***

| ICD-10 disorders | PGS | OR | SE | p-value | Lower CI | Upper CI |
| --- | --- | --- | --- | --- | --- | --- |
| F1 | PGS-MDD top 5% vs. bottom 5% | 1.89 | 0.29 | 2.64x10-02 | 1.09 | 3.37 |
| F1 | PGS-ADHD top 5% vs. bottom 5% | 2.51 | 0.29 | 1.38x10-03 | 1.45 | 4.51 |
| F1 | PGS-SCZ top 5% vs. bottom 5% | 1.70 | 0.29 | 6.53x10-02 | 0.97 | 3.03 |
| F1 | PGS-BD top 5% vs. bottom 5% | 1.60 | 0.28 | 9.98x10-02 | 0.92 | 2.83 |
| F1 | PGS-AN top 5% vs. bottom 5% | 1.00 | 0.30 | 9.92x10-01 | 0.55 | 1.83 |
| F1 | PGS-ASD top 5% vs. bottom 5% | 1.75 | 0.31 | 6.82x10-02 | 0.97 | 3.26 |
| F1 | PGS-MDD top 5% vs. bottom 95% | 1.43 | 0.18 | 4.86x10-02 | 0.99 | 2.01 |
| F1 | PGS-ADHD top 5% vs. bottom 95% | 1.77 | 0.17 | 8.79x10-04 | 1.25 | 2.46 |
| F1 | PGS-SCZ top 5% vs. bottom 95% | 1.29 | 0.18 | 1.69x10-01 | 0.88 | 1.83 |
| F1 | PGS-BD top 5% vs. bottom 95% | 1.51 | 0.18 | 1.96x10-02 | 1.05 | 2.11 |
| F1 | PGS-AN top 5% vs. bottom 95% | 0.94 | 0.21 | 7.87x10-01 | 0.61 | 1.40 |
| F1 | PGS-ASD top 5% vs. bottom 95% | 1.20 | 0.20 | 3.52x10-01 | 0.80 | 1.73 |
| F1 | PGS-MDD top 5% vs. bottom 50% | 1.62 | 0.19 | 9.57x10-03 | 1.11 | 2.32 |
| F1 | PGS-ADHD top 5% vs. bottom 50% | 2.14 | 0.18 | 2.40x10-05 | 1.49 | 3.02 |
| F1 | PGS-SCZ top 5% vs. bottom 50% | 1.55 | 0.19 | 2.35x10-02 | 1.05 | 2.24 |
| F1 | PGS-BD top 5% vs. bottom 50% | 1.51 | 0.18 | 2.40x10-02 | 1.04 | 2.14 |
| F1 | PGS-AN top 5% vs. bottom 50% | 0.90 | 0.21 | 6.38x10-01 | 0.58 | 1.35 |
| F1 | PGS-ASD top 5% vs. bottom 50% | 1.11 | 0.20 | 6.09x10-01 | 0.74 | 1.62 |

***ICD-10 F2 disorders – Schizophrenia. schizotypal and delusional disorder***

| ICD-10 disorders | PGS | OR | SE | p-value | Lower CI | Upper CI |
| --- | --- | --- | --- | --- | --- | --- |
| F2 | PGS-MDD top 5% vs. bottom 5% | 1.98 | 0.29 | 1.99x10-02 | 1.13 | 3.58 |
| F2 | PGS-ADHD top 5% vs. bottom 5% | 1.01 | 0.28 | 9.70x10-01 | 0.58 | 1.76 |
| F2 | PGS-SCZ top 5% vs. bottom 5% | 2.20 | 0.31 | 1.01x10-02 | 1.22 | 4.06 |
| F2 | PGS-BD top 5% vs. bottom 5% | 1.36 | 0.27 | 2.54x10-01 | 0.80 | 2.33 |
| F2 | PGS-AN top 5% vs. bottom 5% | 2.31 | 0.28 | 3.25x10-03 | 1.34 | 4.10 |
| F2 | PGS-ASD top 5% vs. bottom 5% | 2.01 | 0.30 | 1.99x10-02 | 1.13 | 3.68 |
| F2 | PGS-MDD top 5% vs. bottom 95% | 1.60 | 0.18 | 8.50x10-03 | 1.11 | 2.24 |
| F2 | PGS-ADHD top 5% vs. bottom 95% | 1.07 | 0.20 | 7.51x10-01 | 0.71 | 1.56 |
| F2 | PGS-SCZ top 5% vs. bottom 95% | 1.31 | 0.19 | 1.47x10-01 | 0.90 | 1.87 |
| F2 | PGS-BD top 5% vs. bottom 95% | 1.32 | 0.19 | 1.36x10-01 | 0.90 | 1.89 |
| F2 | PGS-AN top 5% vs. bottom 95% | 1.89 | 0.17 | 1.83x10-04 | 1.34 | 2.61 |
| F2 | PGS-ASD top 5% vs. bottom 95% | 1.29 | 0.19 | 1.81x10-01 | 0.88 | 1.85 |
| F2 | PGS-MDD top 5% vs. bottom 50% | 1.55 | 0.18 | 1.75x10-02 | 1.07 | 2.21 |
| F2 | PGS-ADHD top 5% vs. bottom 50% | 1.12 | 0.21 | 5.98x10-01 | 0.73 | 1.66 |
| F2 | PGS-SCZ top 5% vs. bottom 50% | 1.59 | 0.20 | 1.89x10-02 | 1.07 | 2.31 |
| F2 | PGS-BD top 5% vs. bottom 50% | 1.46 | 0.19 | 5.38x10-02 | 0.98 | 2.11 |
| F2 | PGS-AN top 5% vs. bottom 50% | 1.84 | 0.17 | 4.34x10-04 | 1.30 | 2.56 |
| F2 | PGS-ASD top 5% vs. bottom 50% | 1.48 | 0.20 | 4.38x10-02 | 1.00 | 2.16 |

***ICD-10 F4 disorders – Neurotic, stress-related and somatoform disorders***

| ICD-10 disorders | PGS | OR | SE | p-value | Lower CI | Upper CI |
| --- | --- | --- | --- | --- | --- | --- |
| F4 | PGS-MDD top 5% vs. bottom 5% | 1.95 | 0.18 | 1.83x10-04 | 1.38 | 2.79 |
| F4 | PGS-ADHD top 5% vs. bottom 5% | 1.37 | 0.18 | 7.77x10-02 | 0.97 | 1.95 |
| F4 | PGS-SCZ top 5% vs. bottom 5% | 1.10 | 0.17 | 5.72x10-01 | 0.78 | 1.55 |
| F4 | PGS-BD top 5% vs. bottom 5% | 1.20 | 0.18 | 2.92x10-01 | 0.85 | 1.70 |
| F4 | PGS-AN top 5% vs. bottom 5% | 1.00 | 0.18 | 9.88x10-01 | 0.71 | 1.42 |
| F4 | PGS-ASD top 5% vs. bottom 5% | 1.03 | 0.17 | 8.47x10-01 | 0.74 | 1.45 |
| F4 | PGS-MDD top 5% vs. bottom 95% | 1.40 | 0.12 | 4.18x10-03 | 1.11 | 1.77 |
| F4 | PGS-ADHD top 5% vs. bottom 95% | 1.07 | 0.12 | 5.95x10-01 | 0.83 | 1.36 |
| F4 | PGS-SCZ top 5% vs. bottom 95% | 1.18 | 0.12 | 1.66x10-01 | 0.93 | 1.50 |
| F4 | PGS-BD top 5% vs. bottom 95% | 1.23 | 0.12 | 8.42x10-02 | 0.97 | 1.56 |
| F4 | PGS-AN top 5% vs. bottom 95% | 0.92 | 0.13 | 5.05x10-01 | 0.71 | 1.17 |
| F4 | PGS-ASD top 5% vs. bottom 95% | 1.04 | 0.12 | 7.35x10-01 | 0.81 | 1.33 |
| F4 | PGS-MDD top 5% vs. bottom 50% | 1.51 | 0.12 | 7.06x10-04 | 1.19 | 1.92 |
| F4 | PGS-ADHD top 5% vs. bottom 50% | 1.14 | 0.13 | 2.86x10-01 | 0.89 | 1.46 |
| F4 | PGS-SCZ top 5% vs. bottom 50% | 1.23 | 0.13 | 9.34x10-02 | 0.96 | 1.58 |
| F4 | PGS-BD top 5% vs. bottom 50% | 1.22 | 0.12 | 1.05x10-01 | 0.96 | 1.56 |
| F4 | PGS-AN top 5% vs. bottom 50% | 0.96 | 0.13 | 7.53x10-01 | 0.74 | 1.24 |
| F4 | PGS-ASD top 5% vs. bottom 50% | 1.04 | 0.13 | 7.41x10-01 | 0.81 | 1.33 |

***ICD-10 F6 disorders - Personality disorder***

| ICD-10 disorders | PGS | OR | SE | p-value | Lower CI | Upper CI |
| --- | --- | --- | --- | --- | --- | --- |
| F6 | PGS-MDD top 5% vs. bottom 5% | 1.40 | 0.20 | 8.96x10-02 | 0.95 | 2.07 |
| F6 | PGS-ADHD top 5% vs. bottom 5% | 1.76 | 0.20 | 5.52x10-03 | 1.19 | 2.64 |
| F6 | PGS-SCZ top 5% vs. bottom 5% | 1.09 | 0.20 | 6.70x10-01 | 0.74 | 1.61 |
| F6 | PGS-BD top 5% vs. bottom 5% | 1.21 | 0.21 | 3.57x10-01 | 0.80 | 1.83 |
| F6 | PGS-AN top 5% vs. bottom 5% | 1.13 | 0.19 | 5.20x10-01 | 0.78 | 1.66 |
| F6 | PGS-ASD top 5% vs. bottom 5% | 1.42 | 0.20 | 7.93x10-02 | 0.96 | 2.12 |
| F6 | PGS-MDD top 5% vs. bottom 95% | 1.21 | 0.14 | 1.54x10-01 | 0.92 | 1.58 |
| F6 | PGS-ADHD top 5% vs. bottom 95% | 1.32 | 0.13 | 4.08x10-02 | 1.01 | 1.70 |
| F6 | PGS-SCZ top 5% vs. bottom 95% | 1.09 | 0.14 | 5.34x10-01 | 0.82 | 1.43 |
| F6 | PGS-BD top 5% vs. bottom 95% | 1.03 | 0.14 | 8.31x10-01 | 0.77 | 1.36 |
| F6 | PGS-AN top 5% vs. bottom 95% | 1.22 | 0.14 | 1.49x10-01 | 0.93 | 1.58 |
| F6 | PGS-ASD top 5% vs. bottom 95% | 1.17 | 0.14 | 2.52x10-01 | 0.89 | 1.52 |
| F6 | PGS-MDD top 5% vs. bottom 50% | 1.41 | 0.14 | 1.40x10-02 | 1.07 | 1.85 |
| F6 | PGS-ADHD top 5% vs. bottom 50% | 1.40 | 0.14 | 1.40x10-02 | 1.07 | 1.83 |
| F6 | PGS-SCZ top 5% vs. bottom 50% | 1.18 | 0.14 | 2.42x10-01 | 0.89 | 1.56 |
| F6 | PGS-BD top 5% vs. bottom 50% | 1.04 | 0.15 | 7.80x10-01 | 0.77 | 1.38 |
| F6 | PGS-AN top 5% vs. bottom 50% | 1.23 | 0.14 | 1.34x10-01 | 0.93 | 1.61 |
| F6 | PGS-ASD top 5% vs. bottom 50% | 1.18 | 0.14 | 2.44x10-01 | 0.89 | 1.54 |

***ICD-10 F90-F98 disorders - Behavioural and emotional disorders with onset usually occurring in childhood and adolescence***

| ICD-10 disorders | PGS | OR | SE | p-value | Lower CI | Upper CI |
| --- | --- | --- | --- | --- | --- | --- |
| F90-F98 | PGS-MDD top 5% vs. bottom 5% | 1.67 | 0.27 | 5.84x10-02 | 0.99 | 2.86 |
| F90-F98 | PGS-ADHD top 5% vs. bottom 5% | 3.42 | 0.29 | 2.52x10-05 | 1.97 | 6.21 |
| F90-F98 | PGS-SCZ top 5% vs. bottom 5% | 1.06 | 0.30 | 8.59x10-01 | 0.58 | 1.92 |
| F90-F98 | PGS-BD top 5% vs. bottom 5% | 0.82 | 0.30 | 5.06x10-01 | 0.45 | 1.48 |
| F90-F98 | PGS-AN top 5% vs. bottom 5% | 1.28 | 0.30 | 4.19x10-01 | 0.71 | 2.34 |
| F90-F98 | PGS-ASD top 5% vs. bottom 5% | 1.48 | 0.30 | 1.92x10-01 | 0.83 | 2.69 |
| F90-F98 | PGS-MDD top 5% vs. bottom 95% | 1.59 | 0.18 | 8.21x10-03 | 1.11 | 2.23 |
| F90-F98 | PGS-ADHD top 5% vs. bottom 95% | 2.12 | 0.16 | 2.96x10-06 | 1.53 | 2.89 |
| F90-F98 | PGS-SCZ top 5% vs. bottom 95% | 0.90 | 0.21 | 6.06x10-01 | 0.58 | 1.33 |
| F90-F98 | PGS-BD top 5% vs. bottom 95% | 0.91 | 0.21 | 6.64x10-01 | 0.59 | 1.36 |
| F90-F98 | PGS-AN top 5% vs. bottom 95% | 1.03 | 0.21 | 8.80x10-01 | 0.68 | 1.52 |
| F90-F98 | PGS-ASD top 5% vs. bottom 95% | 1.09 | 0.20 | 6.54x10-01 | 0.73 | 1.58 |
| F90-F98 | PGS-MDD top 5% vs. bottom 50% | 1.70 | 0.18 | 3.95x10-03 | 1.17 | 2.41 |
| F90-F98 | PGS-ADHD top 5% vs. bottom 50% | 2.74 | 0.17 | 3.20x10-09 | 1.95 | 3.79 |
| F90-F98 | PGS-SCZ top 5% vs. bottom 50% | 0.94 | 0.22 | 7.75x10-01 | 0.60 | 1.41 |
| F90-F98 | PGS-BD top 5% vs. bottom 50% | 0.93 | 0.22 | 7.58x10-01 | 0.60 | 1.41 |
| F90-F98 | PGS-AN top 5% vs. bottom 50% | 1.03 | 0.21 | 9.07x10-01 | 0.66 | 1.53 |
| F90-F98 | PGS-ASD top 5% vs. bottom 50% | 1.16 | 0.20 | 4.69x10-01 | 0.77 | 1.69 |

*Dark grey background represents results significant at the Bonferroni-adjusted alpha level of 0.00083.*

# STable 14. Association between continuous polygenic scores (PGSs) and treatment for other psychiatric disorders in secondary care in the remaining follow-up in individuals in the brief contact class – mutually adjusted models.

***ICD-10 F1 disorders - Mental and behavioural disorder due to psychoative substance use***

| ICD-10 disorders | PGS | OR | SE | p-value | Lower CI | Upper CI |
| --- | --- | --- | --- | --- | --- | --- |
| F1 | PGS-MDD | 1.09 | 0.05 | 7.48x10-02 | 0.99 | 1.20 |
| F1 | PGS-ADHD | 1.30 | 0.05 | 9.31x10-08 | 1.18 | 1.43 |
| F1 | PGS-ASD | 0.89 | 0.05 | 1.98x10-02 | 0.81 | 0.98 |
| F1 | PGS-BD | 1.03 | 0.05 | 6.21x10-01 | 0.93 | 1.13 |
| F1 | PGS-SCZ | 1.13 | 0.05 | 1.41x10-02 | 1.03 | 1.25 |
| F1 | PGS-AN | 0.96 | 0.05 | 3.79x10-01 | 0.88 | 1.05 |

***ICD-10 F2 disorders - Schizophrenia. schizotypal and delusional disorder***

| ICD-10 disorders | PGS | OR | SE | p-value | Lower CI | Upper CI |
| --- | --- | --- | --- | --- | --- | --- |
| F2 | PGS-MDD | 1.02 | 0.05 | 6.78x10-01 | 0.93 | 1.12 |
| F2 | PGS-ADHD | 1.00 | 0.05 | 9.29x10-01 | 0.90 | 1.10 |
| F2 | PGS-ASD | 1.11 | 0.05 | 3.68x10-02 | 1.01 | 1.22 |
| F2 | PGS-BD | 0.94 | 0.05 | 2.11x10-01 | 0.85 | 1.04 |
| F2 | PGS-SCZ | 1.26 | 0.05 | 5.11x10-06 | 1.14 | 1.40 |
| F2 | PGS-AN | 1.07 | 0.05 | 1.41x10-01 | 0.98 | 1.17 |

***ICD-10 F4 disorders - Neurotic. stress-related and somatoform disorders***

| ICD-10 disorders | PGS | OR | SE | p-value | Lower CI | Upper CI |
| --- | --- | --- | --- | --- | --- | --- |
| F4 | PGS-MDD | 1.11 | 0.03 | 2.97x10-04 | 1.05 | 1.18 |
| F4 | PGS-ADHD | 1.07 | 0.03 | 1.47x10-02 | 1.01 | 1.14 |
| F4 | PGS-ASD | 0.94 | 0.03 | 4.76x10-02 | 0.89 | 1.00 |
| F4 | PGS-BD | 1.01 | 0.03 | 7.70x10-01 | 0.95 | 1.07 |
| F4 | PGS-SCZ | 1.00 | 0.03 | 8.81x10-01 | 0.94 | 1.06 |
| F4 | PGS-AN | 1.05 | 0.03 | 1.05x10-01 | 0.99 | 1.11 |

***ICD-10 F6 disorders - Personality disorder***

| ICD-10 disorders | PGS | OR | SE | p-value | Lower CI | Upper CI |
| --- | --- | --- | --- | --- | --- | --- |
| F6 | PGS-MDD | 1.13 | 0.03 | 3.89x10-04 | 1.06 | 1.20 |
| F6 | PGS-ADHD | 1.09 | 0.03 | 1.31x10-02 | 1.02 | 1.16 |
| F6 | PGS-ASD | 0.97 | 0.03 | 3.74x10-01 | 0.91 | 1.04 |
| F6 | PGS-BD | 0.97 | 0.04 | 4.25x10-01 | 0.91 | 1.04 |
| F6 | PGS-SCZ | 1.04 | 0.04 | 3.30x10-01 | 0.97 | 1.11 |
| F6 | PGS-AN | 1.01 | 0.03 | 6.63x10-01 | 0.95 | 1.08 |

***ICD-10 F90-F98 disorders - Behavioural and emotional disorders with onset usually occurring in childhood and adolescence***

| ICD-10 disorders | PGS | OR | SE | p-value | Lower CI | Upper CI |
| --- | --- | --- | --- | --- | --- | --- |
| F90-F98 | PGS-MDD | 1.04 | 0.05 | 4.45x10-01 | 0.94 | 1.14 |
| F90-F98 | PGS-ADHD | 1.41 | 0.05 | 4.43x10-12 | 1.28 | 1.55 |
| F90-F98 | PGS-ASD | 0.98 | 0.05 | 6.16x10-01 | 0.89 | 1.07 |
| F90-F98 | PGS-BD | 0.95 | 0.05 | 3.11x10-01 | 0.86 | 1.05 |
| F90-F98 | PGS-SCZ | 1.00 | 0.05 | 9.35x10-01 | 0.91 | 1.11 |
| F90-F98 | PGS-AN | 0.99 | 0.05 | 8.73x10-01 | 0.91 | 1.09 |

*Dark grey background represents results significant at the Bonferroni-adjusted alpha level of 0.00083.*

# Stable 15. Associations between parental history of psychiatric disorders and trajectory class membership.

| Trajectory class | Parental history | OR | SE | p-value | Lower CI | Upper CI |
| --- | --- | --- | --- | --- | --- | --- |
| Prolonged initial contact | MDD | 0.94 | 0.07 | 3.91x10-01 | 0.82 | 1.08 |
| Later re-entry | MDD | 1.31 | 0.09 | 3.26x10-03 | 1.09 | 1.57 |
| Persistent contact | MDD | 1.02 | 0.11 | 8.64x10-01 | 0.83 | 1.26 |
| Prolonged initial contact | BD | 0.68 | 0.17 | 1.83x10-02 | 0.49 | 0.94 |
| Later re-entry | BD | 1.22 | 0.20 | 3.18x10-01 | 0.83 | 1.79 |
| Persistent contact | BD | 0.87 | 0.24 | 5.73x10-01 | 0.55 | 1.40 |
| Prolonged initial contact | SCZ spectrum disorders | 0.92 | 0.14 | 5.45x10-01 | 0.70 | 1.21 |
| Later re-entry | SCZ spectrum disorders | 1.10 | 0.19 | 6.32x10-01 | 0.76 | 1.59 |
| Persistent contact | SCZ spectrum disorders | 1.43 | 0.18 | 5.21x10-02 | 1.00 | 2.05 |

*The brief contact class (class 1) was used as the reference category in multinomial regressions. Dark grey background represents results significant at the Bonferroni-adjusted alpha level of 0.00083.*

# STable 16. Associations between parental history of psychiatric disorders and continued treatment in primary care for MDD in the remaining follow-up in individuals in the brief contact class.

***Any time after final discharge***

| Antidepressants | Parental history | OR | SE | p-value | Lower CI | Upper CI |
| --- | --- | --- | --- | --- | --- | --- |
| Any time | MDD | 1.21 | 0.08 | 1.55x10-02 | 1.04 | 1.41 |
| Any time | BD | 0.90 | 0.16 | 5.23x10-01 | 0.67 | 1.24 |
| Any time | SCZ spectrum disorders | 1.00 | 0.15 | 9.81x10-01 | 0.75 | 1.36 |

***+6 months after final discharge***

| Antidepressants | Parental history | OR | SE | p-value | Lower CI | Upper CI |
| --- | --- | --- | --- | --- | --- | --- |
| +6 months | MDD | 1.24 | 0.07 | 2.13x10-03 | 1.08 | 1.43 |
| +6 months | BD | 0.92 | 0.15 | 5.81x10-01 | 0.69 | 1.23 |
| +6 months | SCZ spectrum disorders | 0.99 | 0.14 | 9.59x10-01 | 0.76 | 1.30 |

***+1 year after final discharge***

| Antidepressants | Parental history | OR | SE | p-value | Lower CI | Upper CI |
| --- | --- | --- | --- | --- | --- | --- |
| +1 year | MDD | 1.17 | 0.07 | 2.13x10-02 | 1.02 | 1.34 |
| +1 year | BD | 0.94 | 0.14 | 6.47x10-01 | 0.71 | 1.24 |
| +1 year | SCZ spectrum disorders | 1.00 | 0.13 | 9.82x10-01 | 0.77 | 1.31 |

*Continued treatment in primary care was indexed by redeeming a prescription of antidepressants in the remaining follow-up period after the final discharge from a psychiatric hospital for MDD. Three analyses were conducted: 1) including prescriptions redeemed any time after final discharge, 2) including prescriptions redeemed only 6 months or more after final discharge, and 3) including prescriptions redeemed only 12 months or more after final discharge. Dark grey background represents results significant at the Bonferroni-adjusted alpha level of 0.00083.*

# STable 17. Associations between parental history of psychiatric disorders and treatment for other psychiatric disorders in secondary care in the remaining follow-up in individuals in the brief contact class.

***ICD-10 F1 disorders - Mental and behavioural disorder due to psychoative substance use***

| ICD-10 disorders | Parental history | OR | SE | p-value | Lower CI | Upper CI |
| --- | --- | --- | --- | --- | --- | --- |
| F1 | MDD | 1.26 | 0.12 | 4.51x10-02 | 1.00 | 1.57 |
| F1 | BD | 1.09 | 0.25 | 7.39x10-01 | 0.64 | 1.74 |
| F1 | SCZ spectrum disorders | 1.55 | 0.21 | 3.40x10-02 | 1.01 | 2.28 |

***ICD-10 F2 disorders - Schizophrenia. schizotypal and delusional disorder***

| ICD-10 disorders | Parental history | OR | SE | p-value | Lower CI | Upper CI |
| --- | --- | --- | --- | --- | --- | --- |
| F2 | MDD | 1.07 | 0.11 | 5.27x10-01 | 0.87 | 1.31 |
| F2 | BD | 1.07 | 0.22 | 7.44x10-01 | 0.68 | 1.63 |
| F2 | SCZ spectrum disorders | 1.65 | 0.18 | 5.44x10-03 | 1.14 | 2.32 |

***ICD-10 F4 disorders - Neurotic, stress-related and somatoform disorders***

| ICD-10 disorders | Parental history | OR | SE | p-value | Lower CI | Upper CI |
| --- | --- | --- | --- | --- | --- | --- |
| F4 | MDD | 1.08 | 0.07 | 2.94x10-01 | 0.93 | 1.25 |
| F4 | BD | 0.85 | 0.16 | 3.24x10-01 | 0.61 | 1.17 |
| F4 | SCZ spectrum disorders | 1.50 | 0.14 | 2.90x10-03 | 1.15 | 1.96 |

***ICD-10 F6 disorders - Personality disorder***

| ICD-10 disorders | Parental history | OR | SE | p-value | Lower CI | Upper CI |
| --- | --- | --- | --- | --- | --- | --- |
| F6 | MDD | 1.09 | 0.08 | 3.06x10-01 | 0.92 | 1.28 |
| F6 | BD | 1.05 | 0.18 | 7.68x10-01 | 0.73 | 1.49 |
| F6 | SCZ spectrum disorders | 1.34 | 0.16 | 5.70x10-02 | 0.98 | 1.81 |

***ICD-10 F90-F98 disorders - Behavioural and emotional disorders with onset usually occurring in childhood and adolescence***

| ICD-10 disorders | Parental history | OR | SE | p-value | Lower CI | Upper CI |
| --- | --- | --- | --- | --- | --- | --- |
| F90-F98 | MDD | 1.29 | 0.11 | 2.74x10-02 | 1.02 | 1.61 |
| F90-F98 | BD | 1.83 | 0.21 | 3.51x10-03 | 1.20 | 2.71 |
| F90-F98 | SCZ spectrum disorders | 1.08 | 0.24 | 7.57x10-01 | 0.66 | 1.67 |

*Dark grey background represents results significant at the Bonferroni-adjusted alpha level of 0.00083.*

# STable 18. Associations between continuous polygenic scores (PGSs) and trajectory classes stratified by age-at-index episode.

| AGx10-AT-INDEX EPISODE | TRAJECTORY CLASS | PGS | OR | SE | P-VALUE | LOWER CI | UPPER CI |
| --- | --- | --- | --- | --- | --- | --- | --- |
| 10-15 | Prolonged initial contact | PGS-MDD | 0,88 | 0,05 | 1,48x10-02 | 0,79 | 0,98 |
| 10-15 | Later re-entry | PGS-MDD | 1,03 | 0,08 | 6,78x10-01 | 0,88 | 1,22 |
| 10-15 | Persistent contact | PGS-MDD | 0,92 | 0,07 | 2,59x10-01 | 0,80 | 1,06 |
| 10-15 | Prolonged initial contact | PGS-ADHD | 0,85 | 0,05 | 2,83x10-03 | 0,77 | 0,95 |
| 10-15 | Later re-entry | PGS-ADHD | 1,03 | 0,08 | 6,97x10-01 | 0,88 | 1,21 |
| 10-15 | Persistent contact | PGS-ADHD | 0,81 | 0,07 | 3,78x10-03 | 0,71 | 0,94 |
| 10-15 | Prolonged initial contact | PGSASD_std | 0,98 | 0,05 | 7,53x10-01 | 0,89 | 1,09 |
| 10-15 | Later re-entry | PGSASD_std | 1,03 | 0,08 | 7,58x10-01 | 0,87 | 1,20 |
| 10-15 | Persistent contact | PGSASD_std | 0,91 | 0,07 | 1,79x10-01 | 0,79 | 1,04 |
| 10-15 | Prolonged initial contact | PGS-BD | 1,00 | 0,05 | 9,50x10-01 | 0,90 | 1,12 |
| 10-15 | Later re-entry | PGS-BD | 0,90 | 0,09 | 2,28x10-01 | 0,76 | 1,07 |
| 10-15 | Persistent contact | PGS-BD | 0,92 | 0,07 | 2,70x10-01 | 0,80 | 1,07 |
| 10-15 | Prolonged initial contact | PGS-SCZ | 0,94 | 0,05 | 2,24x10-01 | 0,84 | 1,04 |
| 10-15 | Later re-entry | PGS-SCZ | 1,08 | 0,08 | 3,45x10-01 | 0,92 | 1,27 |
| 10-15 | Persistent contact | PGS-SCZ | 1,00 | 0,07 | 9,83x10-01 | 0,87 | 1,15 |
| 10-15 | Prolonged initial contact | PGS-AN | 1,10 | 0,05 | 5,89x10-02 | 1,00 | 1,22 |
| 10-15 | Later re-entry | PGS-AN | 1,06 | 0,08 | 4,85x10-01 | 0,90 | 1,24 |
| 10-15 | Persistent contact | PGS-AN | 1,25 | 0,07 | 1,56x10-03 | 1,09 | 1,44 |

| AGx10-AT-INDEX EPISODE | TRAJECTORY CLASS | PGS | OR | SE | P-VALUE | LOWER CI | UPPER CI |
| --- | --- | --- | --- | --- | --- | --- | --- |
| 16-20 | Prolonged initial contact | PGS-MDD | 0,95 | 0,04 | 1,31x10-01 | 0,88 | 1,02 |
| 16-20 | Later re-entry | PGS-MDD | 1,07 | 0,05 | 1,76x10-01 | 0,97 | 1,19 |
| 16-20 | Persistent contact | PGS-MDD | 0,92 | 0,06 | 1,76x10-01 | 0,81 | 1,04 |
| 16-20 | Prolonged initial contact | PGS-ADHD | 0,95 | 0,04 | 1,17x10-01 | 0,88 | 1,01 |
| 16-20 | Later re-entry | PGS-ADHD | 0,99 | 0,05 | 9,19x10-01 | 0,90 | 1,10 |
| 16-20 | Persistent contact | PGS-ADHD | 0,84 | 0,06 | 5,03x10-03 | 0,74 | 0,95 |
| 16-20 | Prolonged initial contact | PGSASD_std | 1,05 | 0,04 | 2,09x10-01 | 0,98 | 1,12 |
| 16-20 | Later re-entry | PGSASD_std | 1,10 | 0,05 | 6,72x10-02 | 0,99 | 1,22 |
| 16-20 | Persistent contact | PGSASD_std | 0,96 | 0,06 | 4,74x10-01 | 0,85 | 1,08 |
| 16-20 | Prolonged initial contact | PGS-BD | 1,02 | 0,04 | 5,07x10-01 | 0,95 | 1,10 |
| 16-20 | Later re-entry | PGS-BD | 1,03 | 0,05 | 5,67x10-01 | 0,93 | 1,14 |
| 16-20 | Persistent contact | PGS-BD | 1,05 | 0,06 | 4,54x10-01 | 0,93 | 1,19 |
| 16-20 | Prolonged initial contact | PGS-SCZ | 1,00 | 0,04 | 9,87x10-01 | 0,93 | 1,07 |
| 16-20 | Later re-entry | PGS-SCZ | 1,07 | 0,05 | 2,05x10-01 | 0,96 | 1,18 |
| 16-20 | Persistent contact | PGS-SCZ | 0,98 | 0,06 | 7,15x10-01 | 0,86 | 1,11 |
| 16-20 | Prolonged initial contact | PGS-AN | 1,06 | 0,04 | 8,47x10-02 | 0,99 | 1,14 |
| 16-20 | Later re-entry | PGS-AN | 1,01 | 0,05 | 8,30x10-01 | 0,91 | 1,12 |
| 16-20 | Persistent contact | PGS-AN | 1,13 | 0,06 | 5,24x10-02 | 1,00 | 1,28 |

| AGx10-AT-INDEX EPISODE | TRAJECTORY CLASS | PGS | OR | SE | P-VALUE | LOWER CI | UPPER CI |
| --- | --- | --- | --- | --- | --- | --- | --- |
| 21-25 | Prolonged initial contact | PGS-MDD | 1,04 | 0,05 | 4,19x10-01 | 0,95 | 1,14 |
| 21-25 | Later re-entry | PGS-MDD | 1,16 | 0,07 | 2,10x10-02 | 1,02 | 1,32 |
| 21-25 | Persistent contact | PGS-MDD | 1,06 | 0,08 | 4,62X10-01 | 0,91 | 1,22 |
| 21-25 | Prolonged initial contact | PGS-ADHD | 0,90 | 0,05 | 3,01x10-02 | 0,82 | 0,99 |
| 21-25 | Later re-entry | PGS-ADHD | 0,98 | 0,07 | 7,51x10-01 | 0,86 | 1,11 |
| 21-25 | Persistent contact | PGS-ADHD | 1,13 | 0,08 | 9,68x10-02 | 0,98 | 1,31 |
| 21-25 | Prolonged initial contact | PGSASD_std | 0,99 | 0,05 | 7,98x10-01 | 0,90 | 1,08 |
| 21-25 | Later re-entry | PGSASD_std | 1,09 | 0,07 | 2,04x10-01 | 0,95 | 1,24 |
| 21-25 | Persistent contact | PGSASD_std | 1,00 | 0,08 | 9,95x10-01 | 0,86 | 1,16 |
| 21-25 | Prolonged initial contact | PGS-BD | 1,06 | 0,05 | 2,29x10-01 | 0,97 | 1,16 |
| 21-25 | Later re-entry | PGS-BD | 0,98 | 0,07 | 7,69x10-01 | 0,86 | 1,11 |
| 21-25 | Persistent contact | PGS-BD | 0,95 | 0,07 | 5,22x10-01 | 0,82 | 1,10 |
| 21-25 | Prolonged initial contact | PGS-SCZ | 1,01 | 0,05 | 7,73x10-01 | 0,92 | 1,11 |
| 21-25 | Later re-entry | PGS-SCZ | 0,99 | 0,07 | 8,28x10-01 | 0,87 | 1,12 |
| 21-25 | Persistent contact | PGS-SCZ | 1,01 | 0,08 | 9,40x10-01 | 0,87 | 1,17 |
| 21-25 | Prolonged initial contact | PGS-AN | 1,01 | 0,05 | 7,92x10-01 | 0,92 | 1,11 |
| 21-25 | Later re-entry | PGS-AN | 1,01 | 0,07 | 9,37x10-01 | 0,88 | 1,15 |
| 21-25 | Persistent contact | PGS-AN | 0,96 | 0,08 | 5,96x10-01 | 0,83 | 1,12 |

*Dark grey background represents results significant at the Bonferroni-adjusted alpha level of 0.00083.*

# STable 19. Associations between continuous polygenic scores (PGSs) and treatment for other psychiatric disorders in secondary care in the remaining follow-up in individuals in the brief contact class stratified by age-at-index episode.

***ICD-10 F1 disorders - Mental and behavioural disorder due to psychoative substance use***

| Age-at-index episode | ICD-10 disorders | PGS | OR | SE | p-value | Lower CI | Upper CI |
| --- | --- | --- | --- | --- | --- | --- | --- |
| 10-15 | F1 | PGS-MDD | 1.03 | 0.14 | 8.57x10-01 | 0.78 | 1.35 |
| 10-15 | F1 | PGS-ADHD | 1.43 | 0.14 | 8.27x10-03 | 1.10 | 1.87 |
| 10-15 | F1 | PGS-ASD | 0.89 | 0.14 | 4.09x10-01 | 0.68 | 1.17 |
| 10-15 | F1 | PGS-BD | 1.21 | 0.14 | 1.63x10-01 | 0.93 | 1.59 |
| 10-15 | F1 | PGS-SCZ | 1.16 | 0.14 | 2.62x10-01 | 0.89 | 1.52 |
| 10-15 | F1 | PGS-AN | 0.86 | 0.14 | 2.65x10-01 | 0.65 | 1.12 |

| Age-at-index episode | ICD-10 disorders | PGS | OR | SE | p-value | Lower CI | Upper CI |
| --- | --- | --- | --- | --- | --- | --- | --- |
| 16-20 | F1 | PGS-MDD | 1.14 | 0.06 | 3.87x10-02 | 1.01 | 1.29 |
| 16-20 | F1 | PGS-ADHD | 1.45 | 0.06 | 7.82x10-09 | 1.28 | 1.65 |
| 16-20 | F1 | PGS-ASD | 1.01 | 0.06 | 8.51x10-01 | 0.90 | 1.14 |
| 16-20 | F1 | PGS-BD | 1.10 | 0.06 | 1.37x10-01 | 0.97 | 1.24 |
| 16-20 | F1 | PGS-SCZ | 1.19 | 0.06 | 5.29x10-03 | 1.05 | 1.35 |
| 16-20 | F1 | PGS-AN | 1.00 | 0.06 | 9.52x10-01 | 0.89 | 1.13 |

| Age-at-index episode | ICD-10 disorders | PGS | OR | SE | p-value | Lower CI | Upper CI |
| --- | --- | --- | --- | --- | --- | --- | --- |
| 21-25 | F1 | PGS-MDD | 1.23 | 0.07 | 5.09x10-03 | 1.06 | 1.42 |
| 21-25 | F1 | PGS-ADHD | 1.07 | 0.07 | 3.40x10-01 | 0.93 | 1.24 |
| 21-25 | F1 | PGS-ASD | 1.03 | 0.08 | 6.62x10-01 | 0.89 | 1.20 |
| 21-25 | F1 | PGS-BD | 1.12 | 0.07 | 1.38x10-01 | 0.97 | 1.29 |
| 21-25 | F1 | PGS-SCZ | 1.15 | 0.07 | 5.48x10-02 | 1.00 | 1.34 |
| 21-25 | F1 | PGS-AN | 1.00 | 0.08 | 9.62x10-01 | 0.86 | 1.16 |

***ICD-10 F2 disorders - Schizophrenia. schizotypal and delusional disorder***

| Age-at-index episode | ICD-10 disorders | PGS | OR | SE | p-value | Lower CI | Upper CI |
| --- | --- | --- | --- | --- | --- | --- | --- |
| 10-15 | F2 | PGS-MDD | 1.26 | 0.11 | 3.26x10-02 | 1.02 | 1.55 |
| 10-15 | F2 | PGS-ADHD | 1.18 | 0.10 | 1.11x10-01 | 0.96 | 1.44 |
| 10-15 | F2 | PGS-ASD | 1.16 | 0.11 | 1.70x10-01 | 0.94 | 1.43 |
| 10-15 | F2 | PGS-BD | 1.08 | 0.11 | 4.72x10-01 | 0.88 | 1.33 |
| 10-15 | F2 | PGS-SCZ | 1.23 | 0.10 | 4.75x10-02 | 1.00 | 1.50 |
| 10-15 | F2 | PGS-AN | 1.07 | 0.11 | 5.06x10-01 | 0.87 | 1.32 |

| Age-at-index episode | ICD-10 disorders | PGS | OR | SE | p-value | Lower CI | Upper CI |
| --- | --- | --- | --- | --- | --- | --- | --- |
| 16-20 | F2 | PGS-MDD | 1.06 | 0.06 | 3.75x10-01 | 0.93 | 1.20 |
| 16-20 | F2 | PGS-ADHD | 1.00 | 0.06 | 9.54x10-01 | 0.89 | 1.14 |
| 16-20 | F2 | PGS-ASD | 1.13 | 0.06 | 5.05x10-02 | 1.00 | 1.27 |
| 16-20 | F2 | PGS-BD | 1.11 | 0.06 | 9.55x10-02 | 0.98 | 1.26 |
| 16-20 | F2 | PGS-SCZ | 1.30 | 0.06 | 4.69x10-05 | 1.14 | 1.47 |
| 16-20 | F2 | PGS-AN | 1.05 | 0.06 | 4.13x10-01 | 0.93 | 1.19 |

| Age-at-index episode | ICD-10 disorders | PGS | OR | SE | p-value | Lower CI | Upper CI |
| --- | --- | --- | --- | --- | --- | --- | --- |
| 21-25 | F2 | PGS-MDD | 1.09 | 0.08 | 2.94x10-01 | 0.93 | 1.29 |
| 21-25 | F2 | PGS-ADHD | 1.07 | 0.08 | 4.31x10-01 | 0.91 | 1.26 |
| 21-25 | F2 | PGS-ASD | 1.20 | 0.09 | 3.53x10-02 | 1.01 | 1.42 |
| 21-25 | F2 | PGS-BD | 1.01 | 0.08 | 8.91x10-01 | 0.86 | 1.19 |
| 21-25 | F2 | PGS-SCZ | 1.23 | 0.08 | 1.44x10-02 | 1.04 | 1.45 |
| 21-25 | F2 | PGS-AN | 1.27 | 0.09 | 5.55x10-03 | 1.07 | 1.51 |

***ICD-10 F4 disorders - Neurotic, stress-related and somatoform disorders***

| Age-at-index episode | ICD-10 disorders | PGS | OR | SE | p-value | Lower CI | Upper CI |
| --- | --- | --- | --- | --- | --- | --- | --- |
| 10-15 | F4 | PGS-MDD | 1.10 | 0.06 | 1.54x10-01 | 0.97 | 1.24 |
| 10-15 | F4 | PGS-ADHD | 1.03 | 0.06 | 6.76x10-01 | 0.91 | 1.16 |
| 10-15 | F4 | PGS-ASD | 1.12 | 0.06 | 6.51x10-02 | 0.99 | 1.27 |
| 10-15 | F4 | PGS-BD | 0.91 | 0.06 | 1.48x10-01 | 0.80 | 1.03 |
| 10-15 | F4 | PGS-SCZ | 0.94 | 0.06 | 3.40x10-01 | 0.83 | 1.07 |
| 10-15 | F4 | PGS-AN | 1.04 | 0.06 | 5.73x10-01 | 0.91 | 1.18 |

| Age-at-index episode | ICD-10 disorders | PGS | OR | SE | p-value | Lower CI | Upper CI |
| --- | --- | --- | --- | --- | --- | --- | --- |
| 16-20 | F4 | PGS-MDD | 1.13 | 0.04 | 1.50x10-03 | 1.05 | 1.23 |
| 16-20 | F4 | PGS-ADHD | 1.12 | 0.04 | 3.45x10-03 | 1.04 | 1.21 |
| 16-20 | F4 | PGS-ASD | 0.98 | 0.04 | 6.47x10-01 | 0.91 | 1.06 |
| 16-20 | F4 | PGS-BD | 1.03 | 0.04 | 4.08x10-01 | 0.96 | 1.12 |
| 16-20 | F4 | PGS-SCZ | 1.04 | 0.04 | 3.53x10-01 | 0.96 | 1.12 |
| 16-20 | F4 | PGS-AN | 1.08 | 0.04 | 5.66x10-02 | 1.00 | 1.16 |

| Age-at-index episode | ICD-10 disorders | PGS | OR | SE | p-value | Lower CI | Upper CI |
| --- | --- | --- | --- | --- | --- | --- | --- |
| 21-25 | F4 | PGS-MDD | 1.16 | 0.05 | 2.38x10-03 | 1.05 | 1.27 |
| 21-25 | F4 | PGS-ADHD | 1.05 | 0.05 | 2.86x10-01 | 0.96 | 1.16 |
| 21-25 | F4 | PGS-ASD | 0.93 | 0.05 | 1.62x10-01 | 0.85 | 1.03 |
| 21-25 | F4 | PGS-BD | 1.12 | 0.05 | 1.57x10-02 | 1.02 | 1.24 |
| 21-25 | F4 | PGS-SCZ | 1.06 | 0.05 | 2.26x10-01 | 0.96 | 1.17 |
| 21-25 | F4 | PGS-AN | 1.05 | 0.05 | 3.28x10-01 | 0.95 | 1.16 |

***ICD-10 F6 disorders - Personality disorder***

| Age-at-index episode | ICD-10 disorders | PGS | OR | SE | p-value | Lower CI | Upper CI |
| --- | --- | --- | --- | --- | --- | --- | --- |
| 10-15 | F6 | PGS-MDD | 1.10 | 0.09 | 2.57x10-01 | 0.93 | 1.31 |
| 10-15 | F6 | PGS-ADHD | 1.03 | 0.08 | 6.88x10-01 | 0.88 | 1.22 |
| 10-15 | F6 | PGS-ASD | 1.21 | 0.09 | 2.86x10-02 | 1.02 | 1.44 |
| 10-15 | F6 | PGS-BD | 1.01 | 0.09 | 8.77x10-01 | 0.85 | 1.20 |
| 10-15 | F6 | PGS-SCZ | 0.98 | 0.09 | 8.19x10-01 | 0.83 | 1.16 |
| 10-15 | F6 | PGS-AN | 1.12 | 0.09 | 1.92x10-01 | 0.94 | 1.33 |

| Age-at-index episode | ICD-10 disorders | PGS | OR | SE | p-value | Lower CI | Upper CI |
| --- | --- | --- | --- | --- | --- | --- | --- |
| 16-20 | F6 | PGS-MDD | 1.11 | 0.04 | 1.78x10-02 | 1.02 | 1.20 |
| 16-20 | F6 | PGS-ADHD | 1.13 | 0.04 | 5.26x10-03 | 1.04 | 1.23 |
| 16-20 | F6 | PGS-ASD | 0.97 | 0.04 | 4.48x10-01 | 0.89 | 1.05 |
| 16-20 | F6 | PGS-BD | 1.04 | 0.04 | 3.55x10-01 | 0.96 | 1.13 |
| 16-20 | F6 | PGS-SCZ | 1.08 | 0.04 | 6.57x10-02 | 0.99 | 1.18 |
| 16-20 | F6 | PGS-AN | 0.99 | 0.04 | 8.66x10-01 | 0.91 | 1.08 |

| Age-at-index episode | ICD-10 disorders | PGS | OR | SE | p-value | Lower CI | Upper CI |
| --- | --- | --- | --- | --- | --- | --- | --- |
| 21-25 | F6 | PGS-MDD | 1.23 | 0.06 | 2.19x10-04 | 1.10 | 1.37 |
| 21-25 | F6 | PGS-ADHD | 1.10 | 0.06 | 9.31x10-02 | 0.98 | 1.23 |
| 21-25 | F6 | PGS-ASD | 1.04 | 0.06 | 4.56x10-01 | 0.93 | 1.16 |
| 21-25 | F6 | PGS-BD | 0.99 | 0.05 | 8.96x10-01 | 0.89 | 1.11 |
| 21-25 | F6 | PGS-SCZ | 1.05 | 0.06 | 3.45x10-01 | 0.95 | 1.17 |
| 21-25 | F6 | PGS-AN | 1.07 | 0.06 | 2.05x10-01 | 0.96 | 1.20 |

***ICD-10 F90-F98 disorders – Behavioural and emotional disorders with onset usually occurring in childhood and adolescence***

| Age-at-index episode | ICD-10 disorders | PGS | OR | SE | p-value | Lower CI | Upper CI |
| --- | --- | --- | --- | --- | --- | --- | --- |
| 10-15 | F90-F98 | PGS-MDD | 1.04 | 0.08 | 6.50x10-01 | 0.89 | 1.20 |
| 10-15 | F90-F98 | PGS-ADHD | 1.34 | 0.08 | 1.36x10-04 | 1.15 | 1.55 |
| 10-15 | F90-F98 | PGS-ASD | 1.18 | 0.08 | 3.35x10-02 | 1.01 | 1.37 |
| 10-15 | F90-F98 | PGS-BD | 1.04 | 0.08 | 5.97x10-01 | 0.89 | 1.21 |
| 10-15 | F90-F98 | PGS-SCZ | 1.00 | 0.08 | 9.51x10-01 | 0.86 | 1.16 |
| 10-15 | F90-F98 | PGS-AN | 1.04 | 0.08 | 6.54x10-01 | 0.89 | 1.20 |

| Age-at-index episode | ICD-10 disorders | PGS | OR | SE | p-value | Lower CI | Upper CI |
| --- | --- | --- | --- | --- | --- | --- | --- |
| 16-20 | F90-F98 | PGS-MDD | 1.10 | 0.07 | 1.96x10-01 | 0.95 | 1.26 |
| 16-20 | F90-F98 | PGS-ADHD | 1.47 | 0.07 | 1.79x10-07 | 1.27 | 1.69 |
| 16-20 | F90-F98 | PGS-ASD | 1.01 | 0.07 | 8.62x10-01 | 0.88 | 1.16 |
| 16-20 | F90-F98 | PGS-BD | 0.92 | 0.07 | 2.54x10-01 | 0.80 | 1.06 |
| 16-20 | F90-F98 | PGS-SCZ | 1.00 | 0.07 | 9.89x10-01 | 0.87 | 1.15 |
| 16-20 | F90-F98 | PGS-AN | 0.91 | 0.07 | 1.91x10-01 | 0.79 | 1.05 |

| Age-at-index episode | ICD-10 disorders | PGS | OR | SE | p-value | Lower CI | Upper CI |
| --- | --- | --- | --- | --- | --- | --- | --- |
| 21-25 | F90-F98 | PGS-MDD | 1.30 | 0.09 | 3.68x10-03 | 1.09 | 1.55 |
| 21-25 | F90-F98 | PGS-ADHD | 1.38 | 0.09 | 3.60x10-04 | 1.16 | 1.65 |
| 21-25 | F90-F98 | PGS-ASD | 1.10 | 0.09 | 2.86x10-01 | 0.92 | 1.32 |
| 21-25 | F90-F98 | PGS-BD | 1.01 | 0.09 | 9.22x10-01 | 0.85 | 1.20 |
| 21-25 | F90-F98 | PGS-SCZ | 1.08 | 0.09 | 3.86x10-01 | 0.91 | 1.29 |
| 21-25 | F90-F98 | PGS-AN | 1.09 | 0.09 | 3.37x10-01 | 0.91 | 1.31 |

*Dark grey background represents results significant at the Bonferroni-adjusted alpha level of 0.00083.*

# SFigure 1. Sample selection process


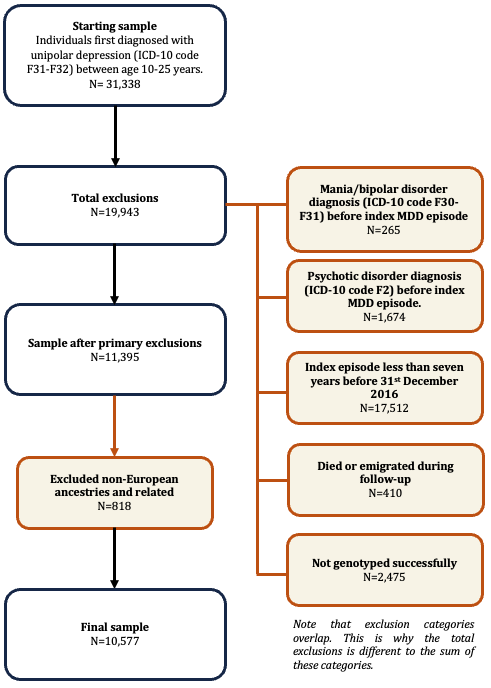


# SFigure 2. Trajectory patterns for Latent Class Growth Analysis (LCGA) models with 1-6 classes.


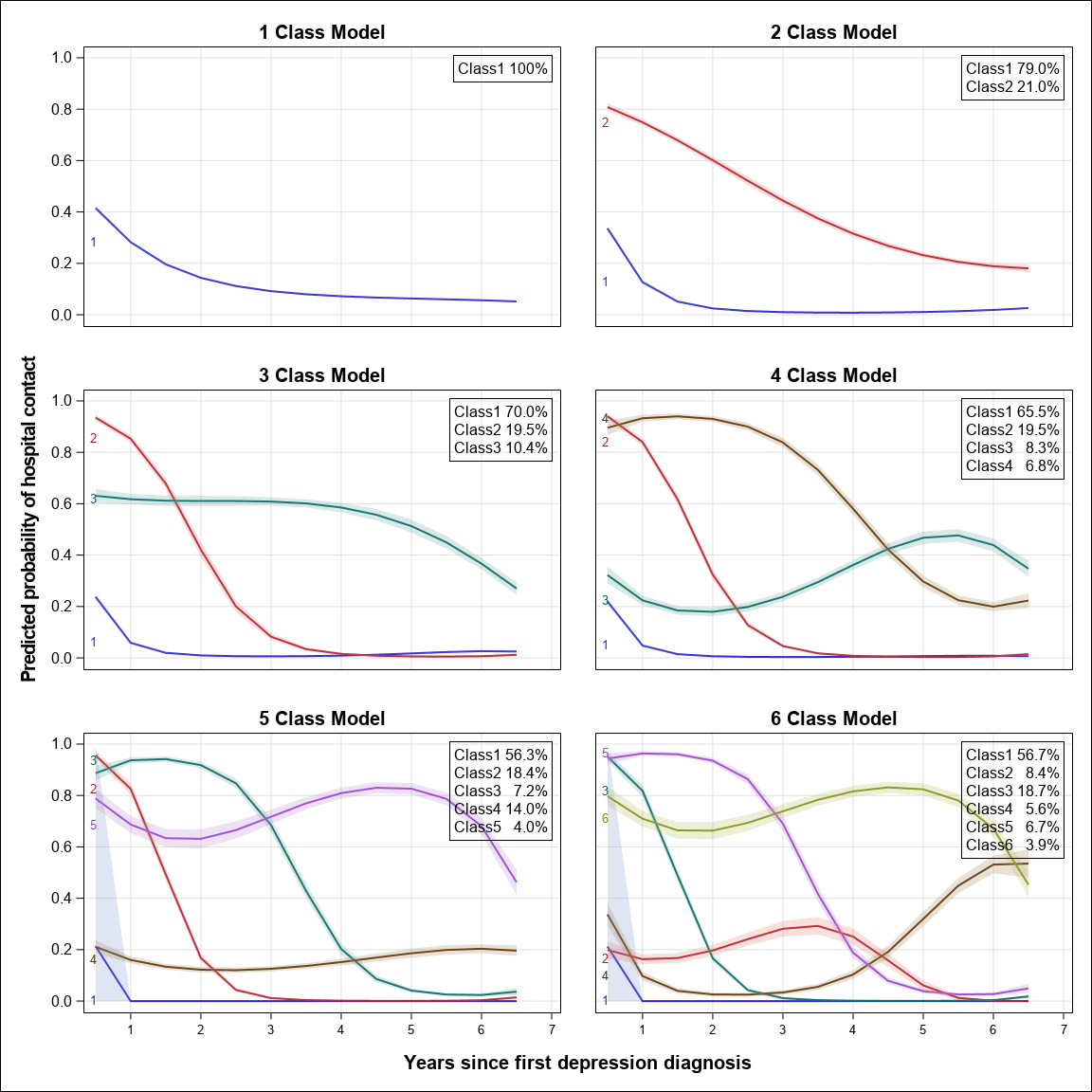


# SFigure 3. Associations between continuous polygenic scores (PGSs) and trajectory classes stratified by age-at-index episode.


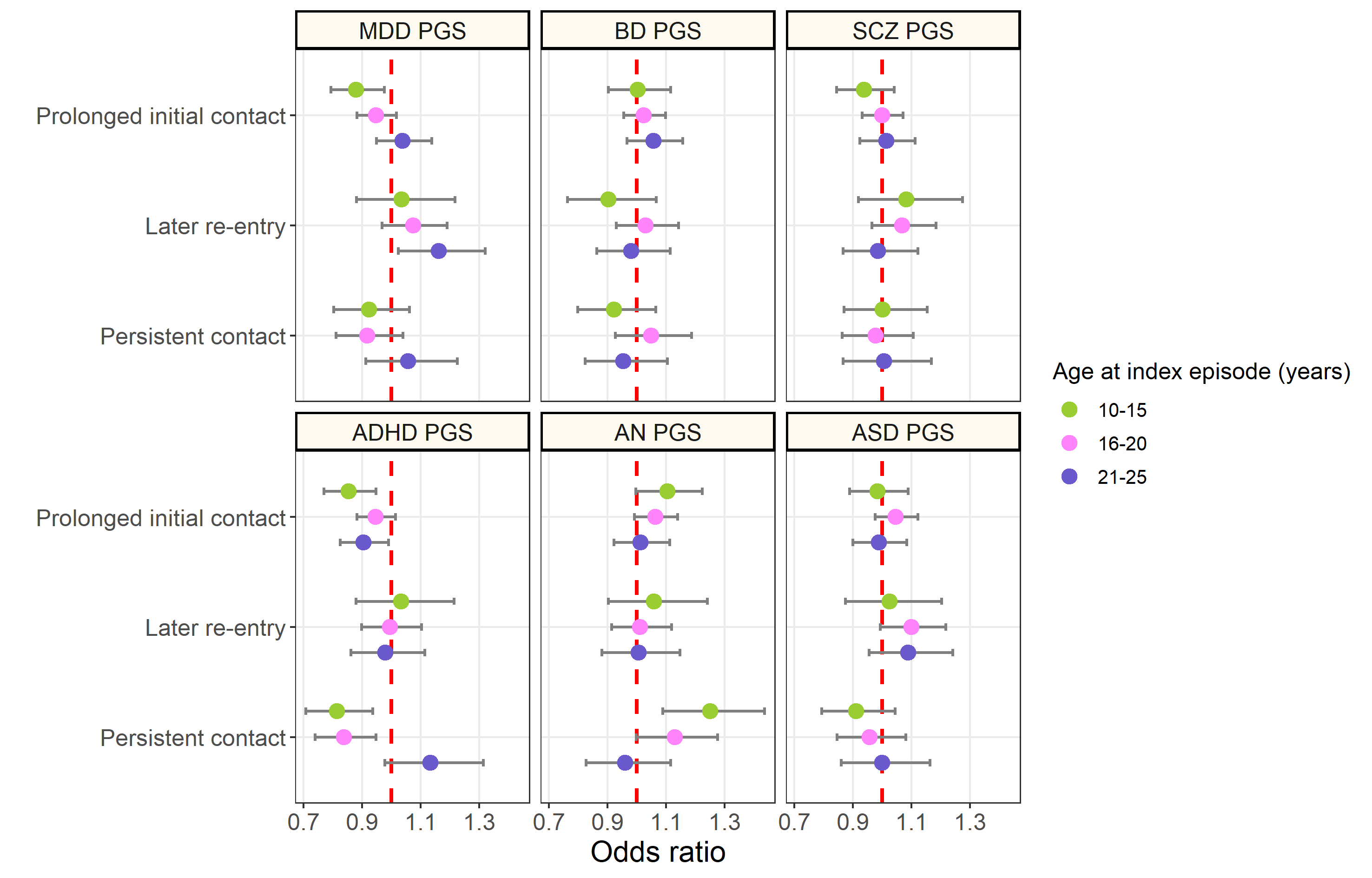


*

*

*

*The brief contact class (class 1) was used as the reference category in the multinomial regressions.*

**Signifiant at the Bonferroni-corrected level p<0.00083.*

# SFigure 4. Associations between continuous polygenic scores (PGSs) and treatment for other psychiatric diagnoses in secondary care in the remaining follow-up in individuals the brief contact class stratified by age-at-index episode.


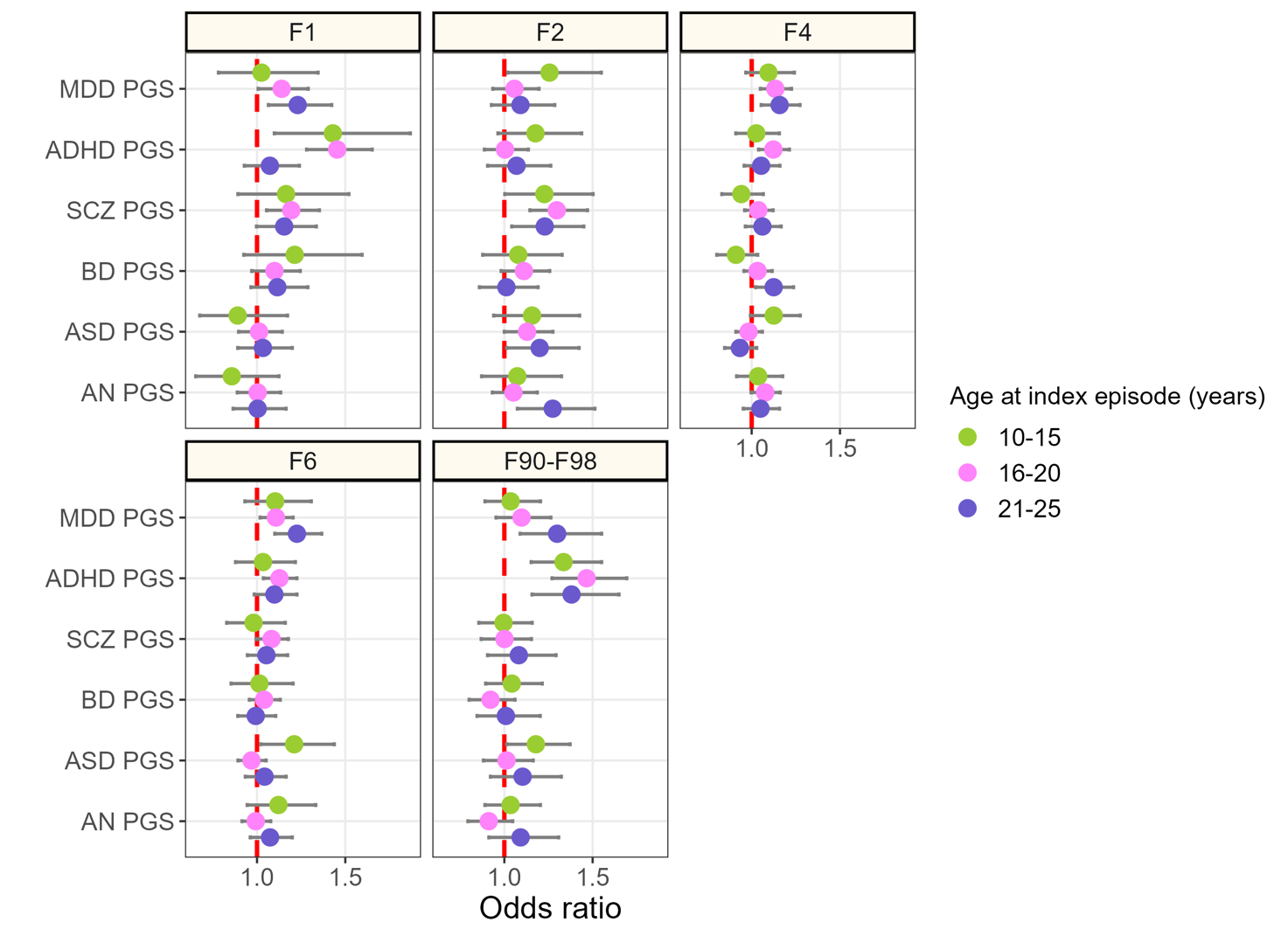


**Significant at the Bonferroni-corrected level p<0.00083.*

# SFigure 5. Associations between parental history of psychiatric disorders and trajectory class membership.


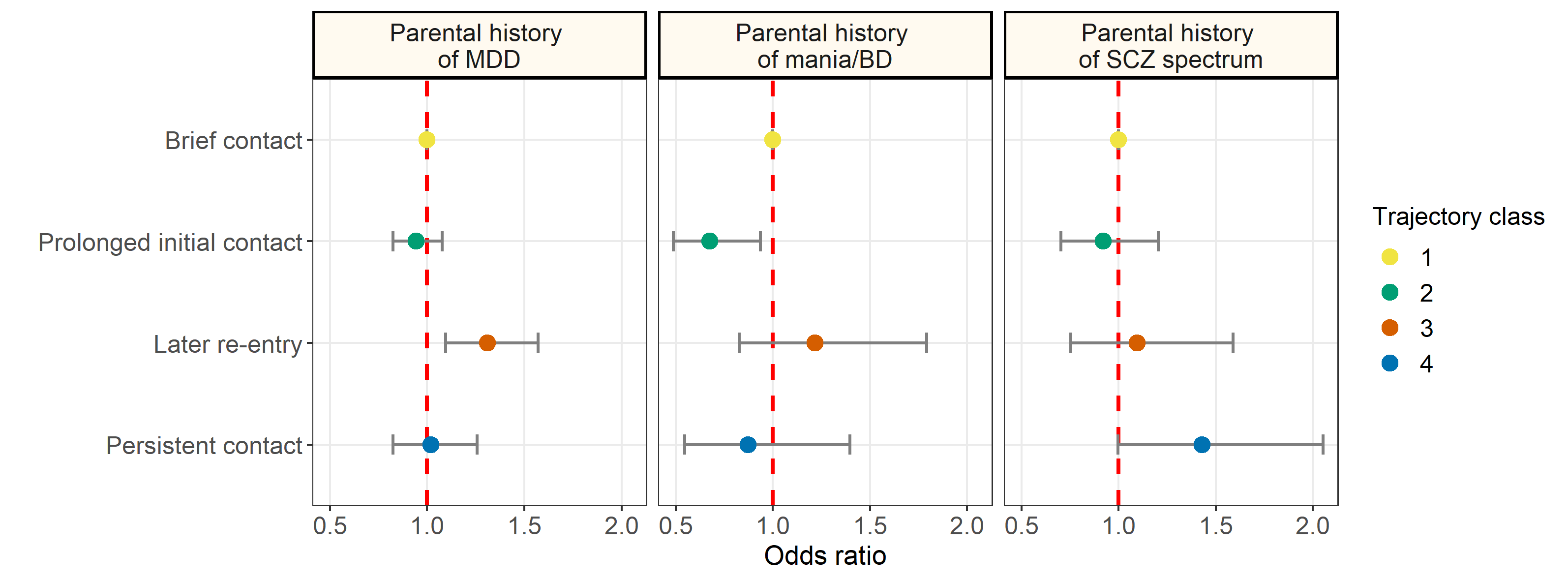


*

*The brief contact class (class 1) was used as the reference category in the multinomial regressions.*

**Significant at the Bonferroni-adjusted level p<.017.*

# SFigure 6. Associations between parental history of psychiatric disorders and continued treatment in primary care in the remaining follow-up in individuals in the brief contact class.


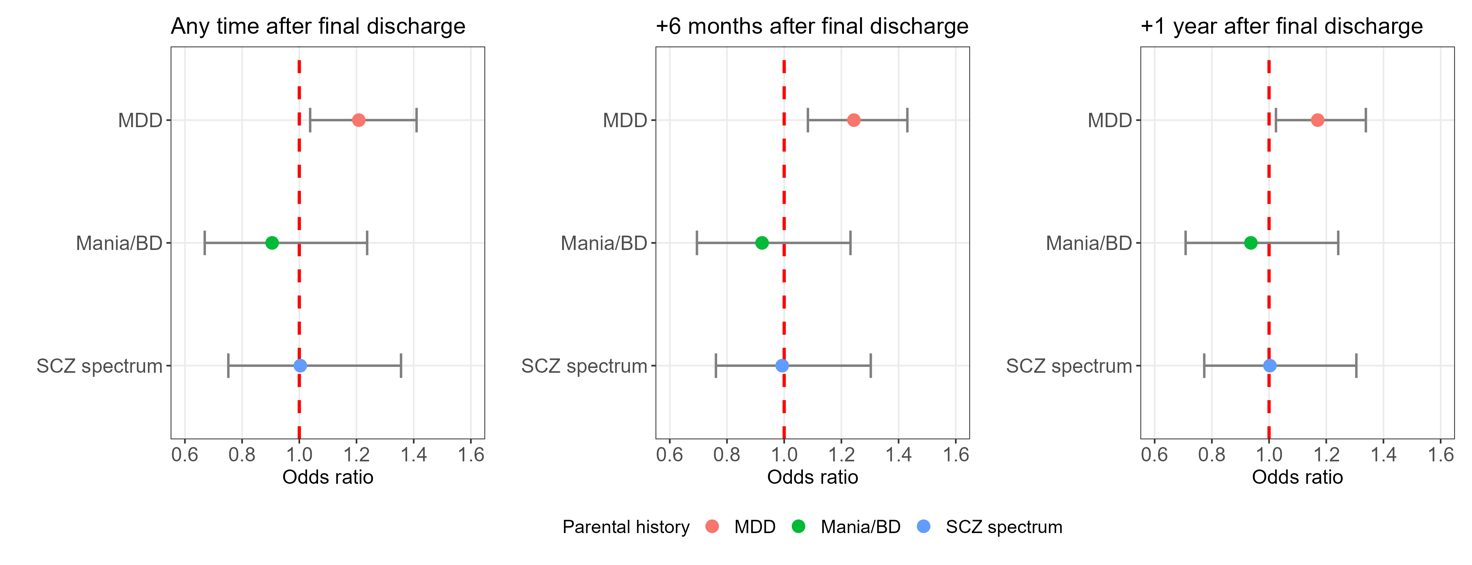


*

*

**Significant at the Bonferroni adjusted alpha level p<.017.*

# SFigure 7. Associations between parental history of psychiatric disorders and treatment for other psychiatric disorders in secondary care in the remaining follow-up in individuals in the brief contact class.

*
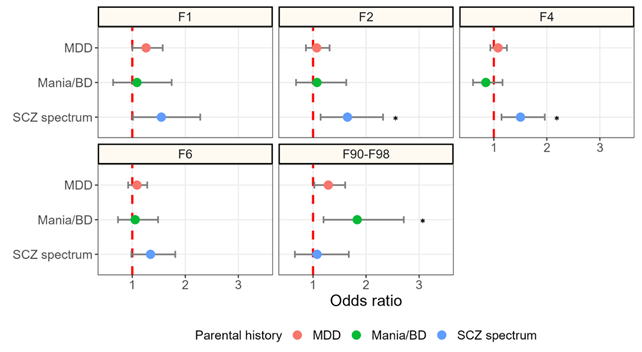
*

** = significant at p<0.017 (Bonferroni-adjusted)*
